# Systematic Investigation into Biochar-Mediated Suppression of Asphalt Rubber Fumes: Inhibition Mechanism, Pollutant Profiles, and Binder Performance

**DOI:** 10.3390/polym18212689

**Published:** 2026-10-29

**Authors:** Zechen Yao, Boqiang Zhang, You Zhou, Shaoshuai Li

**Affiliations:** School of Mechanical and Electrical Engineering, Henan University of Technology, Zhengzhou 450001, China

**Keywords:** asphalt rubber, asphalt fumes, biochar, TVOC, pore structure

## Abstract

Asphalt rubber (AR) is increasingly used as a sustainable pavement binder, but hazardous fumes generated during production constrain its wider application. This study investigated wood biochar (WB), walnut-shell biochar (WSB) and corn-straw biochar (CSB) as sustainable fume-suppressing additives for AR binders. The biochars were characterized by scanning electron microscopy (SEM), elemental analysis (EA), Fourier transform infrared spectroscopy (FTIR), X-ray photoelectron spectroscopy (XPS) and N_2_ physisorption tests, while AR emissions were evaluated through real-time monitoring of total volatile organic compounds (TVOC), H_2_S, SO_2_ and NO_X_, together with thermal desorption-gas chromatography-mass spectrometry (TD-GC-MS) analysis. Conventional binder properties and rheological behavior were also evaluated. The results showed that WSB possessed the most developed hierarchical pore structure, with a Brunauer–Emmett–Teller (BET) surface area of 285.8 m^2^/g and a total pore volume of 0.165 cm^3^/g, achieving the strongest TVOC reduction (65.6%) and the lowest detectable organic-fume complexity. TD-GC-MS analysis showed that WSB mainly reduced the relative responses of aliphatic hydrocarbons and PAH-related hydrocarbons, whereas WB selectively reduced BTEX/alkylbenzenes and S/N-containing responses. CSB, enriched in ash-derived minerals and oxygen-containing groups, exhibited the strongest sulfur-gas suppression, achieving complete SO_2_ elimination and 57.65% H_2_S inhibition, but also induced a redistribution toward oxygenated organics and alicyclic hydrocarbons. Biochar addition generally improved high-temperature stiffness and compatibility. These findings demonstrate feedstock-dependent and pollutant-specific suppression behavior, suggesting that physical adsorption and surface chemical interactions may jointly contribute to fume suppression, and provide guidance for selecting biochar additives for cleaner AR production.

## 1. Introduction

Asphalt rubber (AR), prepared by blending crumb rubber (CR), a recycled polymeric modifier derived from end-of-life tires, with petroleum asphalt, is a sustainable pavement binder that promotes waste rubber recycling while offering improved elastic recovery, rutting resistance, fatigue tolerance, and aging resistance [1,2,3,4,5]. In the wet process, however, AR binder is produced at elevated temperatures to promote rubber swelling, partial degradation and interaction with asphalt fractions. Under these working conditions, light asphalt components and tire additives, including vulcanization residues, plasticizers and antioxidants, can volatilize or decompose, generating AR fumes [6,7,8,9,10,11]. Therefore, large-scale application of AR technology is constrained not only by compatibility and workability, but also by the emission burden associated with production, transportation and paving.

Asphalt fume is a complex multiphase emission system composed of volatile organic compounds (VOCs) and semi-volatile organic compounds (SVOCs), inorganic gaseous pollutants and particulate matter [12,13,14,15,16,17,18,19,20,21]. Compared with base asphalt and other polymer-modified asphalt, AR binder generally exhibits a broader pollutant spectrum, as CR particles introduce sulfur-containing gases, rubber-derived organosulfur compounds and additional aromatic or heterocyclic species [6,7,8,9,10,22,23]. These emissions intensify odor, increase occupational exposure risk and aggravate local atmospheric pollution. As summarized in Table 1, AR fumes contain both conventional asphalt-derived hydrocarbons and additional rubber-derived pollutants. In particular, organosulfur compounds, heterocyclic species and additional PAH-related compounds make AR fume suppression more challenging than fume suppression in conventional asphalt.

Emission mitigation strategies for asphalt materials generally involve either reducing the processing temperature or incorporating additives for fume suppression [30]. The effectiveness of warm-mix approaches in lowering asphalt fume emissions has been reported by previous studies. Yang et al. [11] reported a marked decrease in emissions when AR binder was produced under warm-mix rather than conventional conditions, whereas Rubio et al. [31] showed that asphalt mixtures produced at lower temperatures could significantly reduce atmospheric emissions. For AR binder, however, temperature reduction alone may be insufficient because its fumes are derived not only from volatile asphalt fractions but also from the thermal decomposition of CR particles, vulcanization residues and sulfur-containing constituents.

Another approach is to incorporate active additives into asphalt binder to suppress fume release. Zhang et al. [27] assessed VOC inhibition and rheological evolution of high-content AR binders modified with zeolite, nano-calcium carbonate and activated carbon, proposing suitable process parameters. Li et al. [32] used kaolin and sepiolite as mineral modifiers for AR binder and showed that silicate-based additives can reduce fume emissions through adsorption and physical barrier effects, while also altering the deformation and fatigue properties. Liu et al. [33] investigated montmorillonite nanoclay in AR binder and reported that nanoclay addition suppressed several groups of VOCs, especially alkanes, olefins and aromatic hydrocarbons, owing to its layered structure. Xiao et al. [34] illustrated that activated carbon can decrease VOC release from asphalt binder, mainly through its high surface area and porous structure. Nevertheless, most existing additive-based strategies still focus primarily on VOC reduction, with inherent limitations in pollutant coverage, dosage efficiency and binder compatibility [35,36,37,38,39,40]. Targeted control of multiple pollutant groups in complex AR fumes and the underlying structure-performance relationships remain insufficiently understood.

Biochar is a renewable carbonaceous material with tunable pore structure, surface functionality and mineral components, and has been widely studied as an environmental sorbent [41,42,43,44]. Recently, biochar-based additives have been used to suppress asphalt fumes, showing promising adsorption and emission-reduction potential. Zhou et al. [29] compared different biochars for controlling VOC emissions from asphalt and identified chemical adsorption as an important mechanism under high-temperature conditions. Zhou et al. [45] employed biochars for VOC removal from base asphalt and investigated the adsorption process through experiments and molecular simulations. Zhang et al. [26] investigated functionalized wood flour and bagasse biochar for fume suppression in AR binder, focusing primarily on VOC and H_2_S mitigation. Chen et al. [46] demonstrated that corn-straw biochar could reduce VOC and PAH emissions from base asphalt. Duan et al. [47] further related the fume suppression behavior of cellulose-rich and lignin-rich biochars to their pore structures and oxygen-rich surfaces. Yang et al. [17] reviewed asphalt fume generation and suppression technologies, emphasizing the need for comprehensive evaluation of fume mitigation and binder performance.

Collectively, these studies suggest that biochar-mediated fume suppression is closely related to pore architecture, carbon structure, surface chemistry and mineral constituents, rather than merely the incorporation of biochar as a filler. Nevertheless, systematic comparisons of biochars derived from different feedstocks remain limited, particularly in the more complex AR system. More importantly, how these physicochemical differences influence the suppression of multiple organic and inorganic gaseous pollutants, as well as the compositional redistribution of residual fumes, remains insufficiently understood.

In this study, wood biochar (WB), walnut-shell biochar (WSB), and corn-straw biochar (CSB) were selected as representative fume-suppressing additives for AR binder, as they represented three typical biomass feedstock categories with distinct physicochemical characteristics, allowing a systematic comparison of their pollutant-specific suppression behavior and the associated structure-suppression relationships. Accordingly, the biochars were first characterized by scanning electron microscopy (SEM), elemental analysis (EA), Fourier transform infrared spectroscopy (FTIR), X-ray photoelectron spectroscopy (XPS) and N_2_ physisorption tests. Biochar effects on AR emissions were then evaluated by real-time monitoring of TVOC, H_2_S, SO_2_ and NO_X_, together with Tenax-TA tube sampling and thermal desorption-gas chromatography-mass spectrometry (TD-GC-MS) analysis to identify changes in the chemical composition of fumes and the redistribution of residual organic-fume constituents. Finally, conventional binder properties and rheological behavior were examined to assess the effects of biochar addition on the engineering properties of AR binders in conjunction with fume suppression.

## 2. Materials and Methods

**Figure 1:**
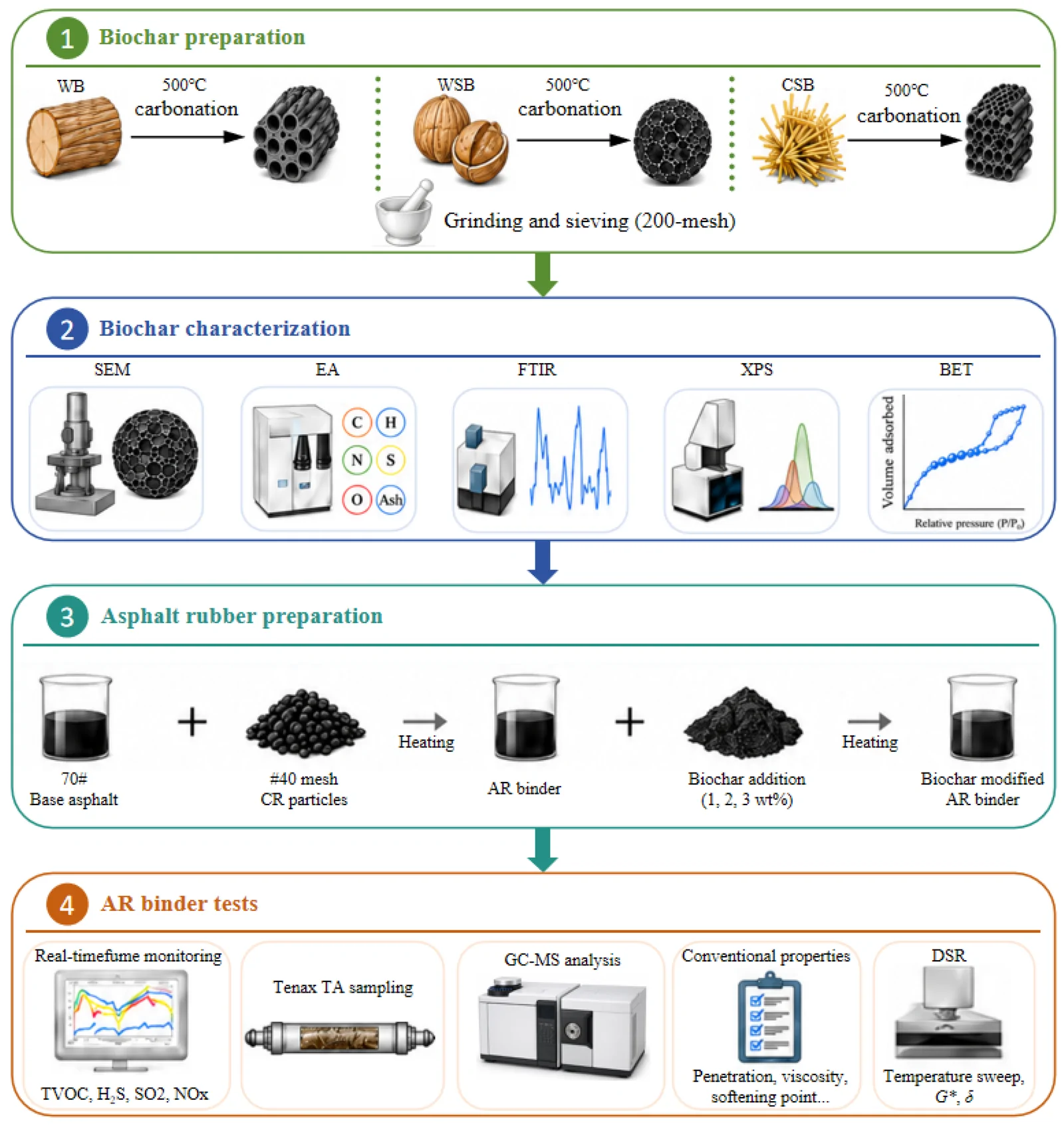
Research scheme.

Figure 1 illustrates the overall research scheme of this study.

### 2.1. Materials and Sample Preparation

As shown in Table 2 and Table 3, a 70-grade base asphalt and 40-mesh CR particles were used to prepare AR binder, which was produced by blending 20 wt% CR with base asphalt at 180 °C. The blend was premixed at low speed for 5 min and then mixed at 1000 rpm for 45 min. WB, WSB and CSB, supplied by Dingyi Company (Zhengzhou, China), were produced from wood, walnut shell and corn straw, respectively. The samples were pyrolyzed under high-purity N_2_ by heating from room temperature to 500 °C at a rate of 10 °C/min and holding at 500 °C for 2 h. All biochars were ground and passed through a 200-mesh sieve (approximately 75 μm aperture size). Biochar-modified AR samples were prepared by adding biochar at dosages of 1, 2 and 3 wt% based on the total mass of AR binder, followed by low-speed premixing for 5 min and high-speed stirring at 1000 rpm for 30 min, as adapted from the literature [47]. The binder samples are illustrated in Table 4.

Real-time emission monitoring was conducted at biochar dosages of 1, 2 and 3 wt% to evaluate dosage-dependent suppression behavior. A representative intermediate dosage of 2 wt% was selected for TD-GC-MS and DSR analyses, primarily to compare the effects of biochar feedstock on organic fume composition and binder rheological properties under identical dosage conditions.

### 2.2. Biochar Characterization

#### 2.2.1. Scanning Electron Microscopy

The surface micromorphology of the biochars was characterized using scanning electron microscopy (SEM-5000X, Guoyiliangzi, Hefei, China). Dry biochar powders of WB, WSB, and CSB were sputter-coated with a thin layer of gold to enhance conductivity and then imaged at an accelerating voltage of 10 kV at magnifications of 1000× and 5000×.

#### 2.2.2. Elemental Analysis

Elemental analysis was conducted using an elemental analyzer (Elementar UNICUBE, Langenselbold, Germany) to determine the C, H, N and S contents of WB, WSB and CSB samples in accordance with NY/T 3498-2019 [49]. Ash content was measured by combustion in a muffle furnace at 750 °C as described in [47], and oxygen content was calculated by difference as O (wt%) = 100 − C − H − N − S − Ash.

#### 2.2.3. Fourier Transform Infrared Spectroscopy

Fourier transform infrared spectroscopy is a widely used technique for analyzing molecular structures and chemical bonds. The chemical structures of WB, WSB, and CSB were characterized using an IRTracer-100 spectrometer (Shimadzu, Kyoto, Japan). FTIR spectra were recorded in the wavenumber range of 500–4000 cm^−1^, with 32 scans and a spectral resolution of 4 cm^−1^.

#### 2.2.4. X-Ray Photoelectron Spectroscopy

X-ray photoelectron spectroscopy was performed using an X-ray photoelectron spectrometer (Thermo Fisher K-Alpha, Waltham, MA, USA) with Al Kα radiation (400 µm spot, 50 eV pass energy, 0.1 eV step, 5 scans) to analyze the surface chemical states of biochar samples. Survey spectra and C 1s/O 1s high-resolution spectra were collected. The high-resolution spectra were fitted to identify the main carbon- and oxygen-containing surface species.

#### 2.2.5. N_2_ Physisorption and Pore Structure Analysis

The pore structures of WB, WSB and CSB were analyzed by N_2_ adsorption–desorption measurements using an Autosorb 6100 gas adsorption analyzer (Anton Paar, Graz, Austria). Before testing, the biochar powders were degassed under vacuum at 150 °C for 12 h to remove moisture and volatile impurities. The adsorption–desorption isotherms were collected at 77 K over a relative pressure range of *P*/*P*_0_ = 0–1. The Brunauer–Emmett–Teller (BET) specific surface area was calculated from the linear region of the adsorption branch, using *P*/*P*_0_ ranges of 0.0757–0.2276, 0.0099–0.0997, and 0.1006–0.2523 for WB, WSB, and CSB, respectively. These sample-specific fitting ranges were selected following GB/T 19587-2017 and the IUPAC recommendations [50,51]. The selected ranges satisfied the BET consistency criteria, including positive *C* constants, monotonically increasing n(1 − *P*/*P*_0_), and monolayer pressures within the corresponding fitting ranges. Detailed BET fitting parameters are provided in Table S1 of the Supplementary Materials. The total pore volume was estimated from the N_2_ uptake near *P*/*P*_0_ = 0.99. The average pore diameter was calculated from the total pore volume and BET surface area. Micropore volume, micropore surface area and external surface area were obtained using the t-plot method, and the mesopore-size distribution was determined by the Barrett–Joyner–Halenda (BJH) method based on the adsorption branch. Pores were classified according to the IUPAC recommendations as micropores (<2 nm), mesopores (2–50 nm) and macropores (>50 nm) [51,52].

### 2.3. Fume Generation, Real-Time Monitoring and Gas Sampling

**Figure 2:**
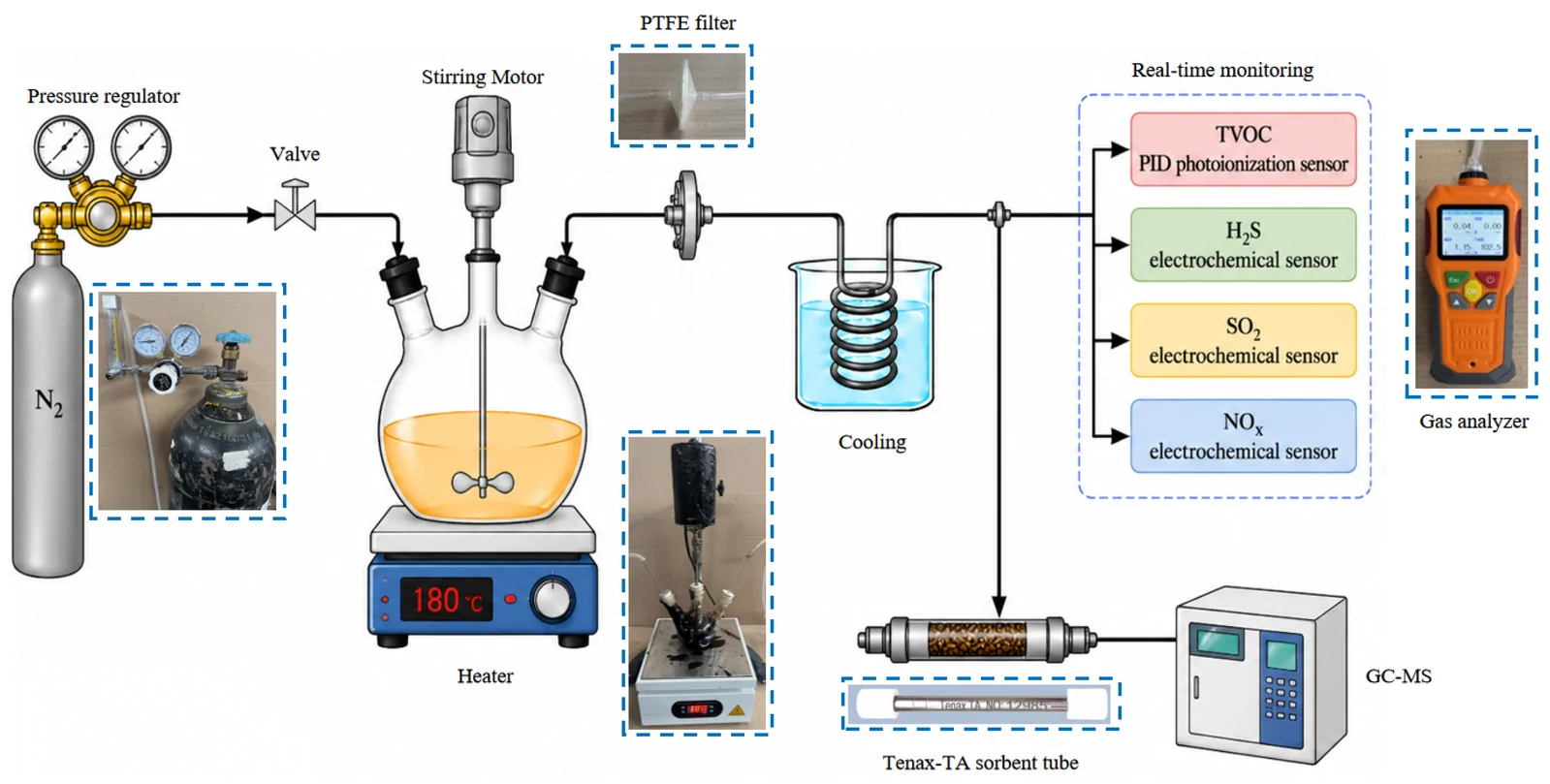
Schematic diagram of the real-time monitoring and TD-GC-MS sampling system.

As shown in Figure 2, a laboratory-scale fume generation system was established to simulate fume release from AR and biochar-modified AR binders as described in [6,47]. A 100 g binder sample was placed in a three-neck flask and maintained at 180 °C using a temperature-controlled heating system. The central neck was fitted with a mechanical stirrer. One side neck was connected to a high-purity N_2_ cylinder via a pressure regulator (Bowei, Linhai, China), while the third neck served as the fume outlet. Before each test, the system was purged with N_2_ for 10 min to eliminate residual atmospheric air and minimize background interference. During fume generation, N_2_ was supplied at 50 mL/min to sweep the evolved fumes out of the flask. The gas stream was first passed through a PTFE membrane filter (0.22 μm, Sheptech, Weifang, China) to remove particulates and then through a cooling unit before entering a four-in-one gas analyzer (PGD3-C-M4, Xin Hairui, Shenzhen, China). Therefore, the measured results represent the particle-filtered gaseous fraction rather than total fume emissions. The gas analyzer was calibrated prior to each test, and real-time concentration profiles were recorded over a period of 1000 s. The operating principles, measurement ranges, and resolutions of the sensors are summarized in Table 5. All real-time monitoring experiments were independently conducted in triplicate, and the results were expressed as the mean ± standard deviation (SD). Two-way analysis of variance (ANOVA) was performed using replicate-level inhibition ratios to evaluate the effects of biochar type and dosage on pollutant suppression, with statistical significance defined as *p* < 0.05.

The inhibition ratio of each gaseous pollutant was calculated as the reduction in the mean pollutant concentration for the biochar-modified AR binder relative to that for the AR binder, following the approach reported by Liang et al. [53]:

(1) $$I_{C}=\frac{{\bar{C}}_{\mathrm{AR}}-{\bar{C}}_{B}}{{\bar{C}}_{\mathrm{AR}}}\times 100\%$$

where *I_C_* is the inhibition ratio for a given pollutant, and ${\bar{C}}_{\mathrm{AR}}$ and ${\bar{C}}_{B}$ represent the mean concentrations of pollutant emitted from the AR binder and biochar-modified AR binder, respectively.

For TD-GC-MS sampling, the gas analyzer was bypassed, and the outlet was connected in series to a Tenax-TA sorbent tube (6.35 mm × 89 mm, Zhejilong, Hefei, China) followed by a 5 V DC air pump (YW11, Yuanwang, Changzhou, China). Under the same heating conditions, N_2_ was continuously supplied to the flask at 50 mL/min as the carrier gas to sweep the evolved fumes, while the downstream air pump was operated at 50 mL/min to draw the fume stream through the Tenax-TA tube. Each sample was collected for 2 min.

The collected Tenax-TA sorbent tubes were analyzed using a thermal desorption system coupled with a GC-MS system (GCMS-TQ8040, Shimadzu, Japan), following ISO 16000-6 [54] and US EPA Method TO-17 [55]. During thermal desorption, the Tenax-TA sorbent tube was initially held at 20 °C for 0.1 min, then heated to 300 °C at 60 °C/min and held for 7.5 min. The desorbed compounds were focused in a cold trap at −150 °C for 0.1 min, followed by heating to 300 °C at 12 °C/min and holding for 3 min. The transfer line temperature was maintained at 300 °C. Chromatographic separation was performed on a DB-5MS capillary column. The oven temperature was initially held at 40 °C for 1 min, increased to 150 °C at 5 °C/min, and then increased to 280 °C at 15 °C/min and held for 5 min. The ion source temperature was set at 230 °C. The results are semi-quantitative, based on total ion current peak areas, and reflect changes in the relative abundance of components. Compounds were tentatively identified by comparing the obtained mass spectra with the NIST/Wiley mass spectral libraries. Only peaks with a spectral similarity index of ≥80 were retained as valid peaks. Unique tentative assignments were determined by removing duplicate compound names among valid peaks. Multiple peaks assigned to the same compound were counted once for this metric but retained separately for peak-area analysis. The tentatively identified organic fume species were classified into six categories: ① aliphatic hydrocarbons, ② alicyclic hydrocarbons, ③ benzene, toluene, ethylbenzene and xylene (BTEX)/alkylbenzenes, ④ polycyclic aromatic hydrocarbon (PAH)-related hydrocarbons, ⑤ oxygenated organics, ⑥ S/N-containing and others.

The relative peak-area inhibition ratio of each organic-fume category was calculated from the summed TIC peak areas of the corresponding compounds, following the relative-reduction approach reported in previous studies [56,57]

(2) $$I_{A}=\frac{A_{\mathrm{AR}}-A_{B}}{A_{\mathrm{AR}}}\times 100\%$$

where *I_A_* is the relative peak-area inhibition ratio, and *A_AR_* and *A_B_* represent the summed TIC peak areas of the tentatively identified compounds within categories for the AR binder and the corresponding biochar-modified AR binder, respectively. Positive values indicate decreases in the relative response, whereas negative values indicate increases.

### 2.4. Binder Characterization

Needle penetration, softening point, rotational viscosity, elastic recovery and storage stability were tested following JTG E20-2011 [58]. Each test was performed in triplicate for each binder formulation. The results satisfied the repeatability limits specified in the corresponding test methods, and the mean values were reported. Dynamic rheological measurements were performed using a DHR-1 rheometer (TA Instruments, New Castle, DE, USA) equipped with 25 mm parallel plates set at a gap of 2 mm. Temperature sweeps were conducted from 60 °C to 85 °C at a fixed angular frequency of 10 rad/s. The complex modulus and phase angle were employed to assess the viscoelastic properties of binders.

## 3. Results and Discussion

### 3.1. Micromorphology of Biochars

**Figure 3:**
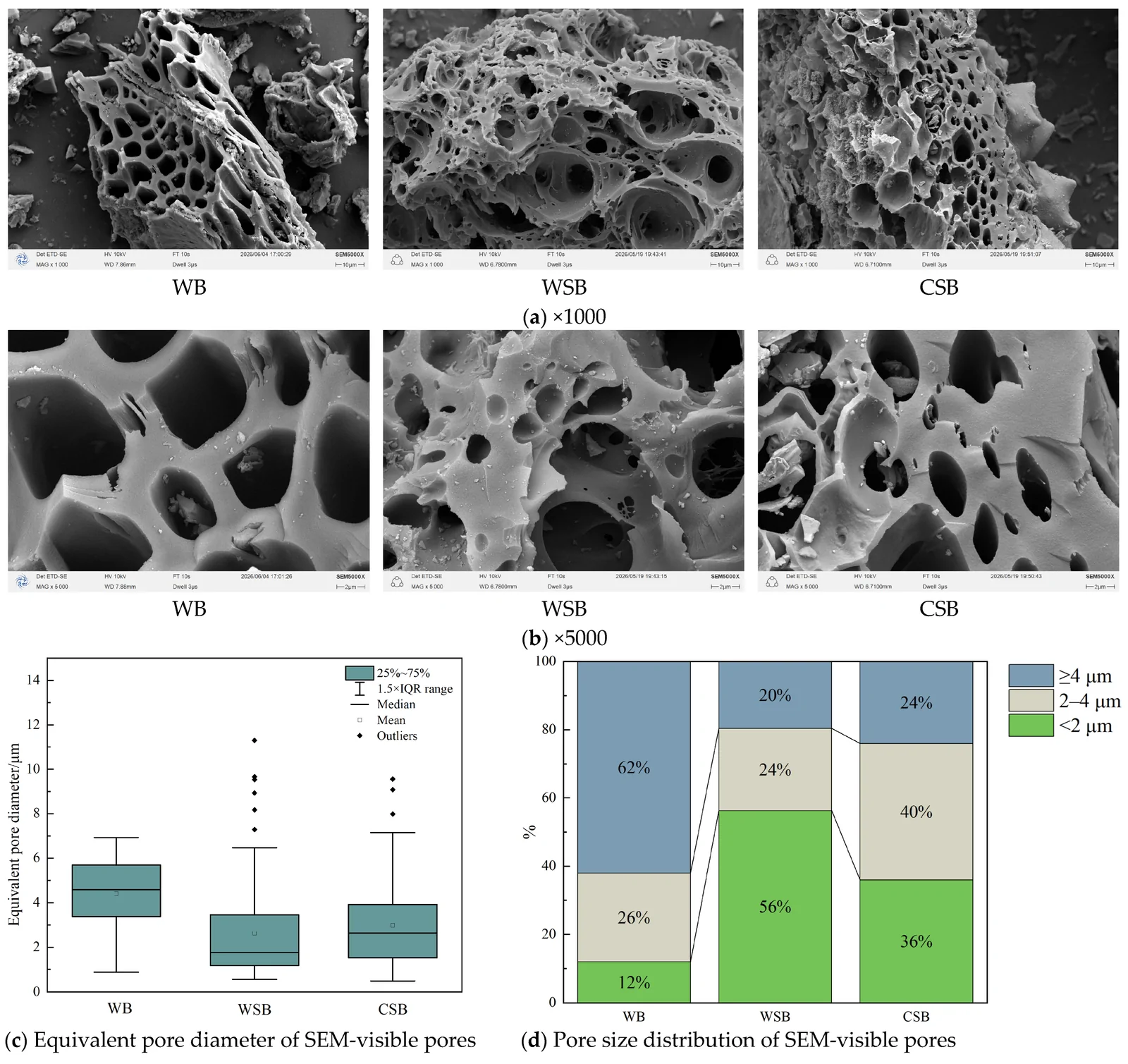
SEM micrographs of biochars.

As shown in Figure 3a,b, WB showed an ordered vascular bundle structure with aligned macropores and dense pore walls with relatively smooth surfaces, but its secondary porosity was limited. WSB presented a more open porous network, characterized by interconnected macropores, rough wrinkled walls and abundant submicron pits, indicating a hierarchical pore structure with higher surface accessibility. In contrast, CSB exhibited honeycomb-shaped pores combined with lamellar carbon sheets, accompanied by pore collapse, wall deformation and brittle fracture edges, suggesting a relatively disordered carbon framework. Additionally, as shown in Figure 3c,d, quantitative analysis of the SEM images using ImageJ (1.54p) revealed that WSB had the smallest median equivalent pore diameter (approximately 1.7 μm) and the highest proportion of pores below 2 μm (56%), compared with WB (12%) and CSB (36%), further confirming its more developed fine-pore structure. These morphological differences may influence fume suppression through pore-mediated physical adsorption and mass transfer, with WSB potentially providing more accessible retention sites for organic fume constituents. The original high-resolution SEM images have been provided in the Supplementary Materials.

### 3.2. Elemental Composition of Biochars

**Figure 4:**
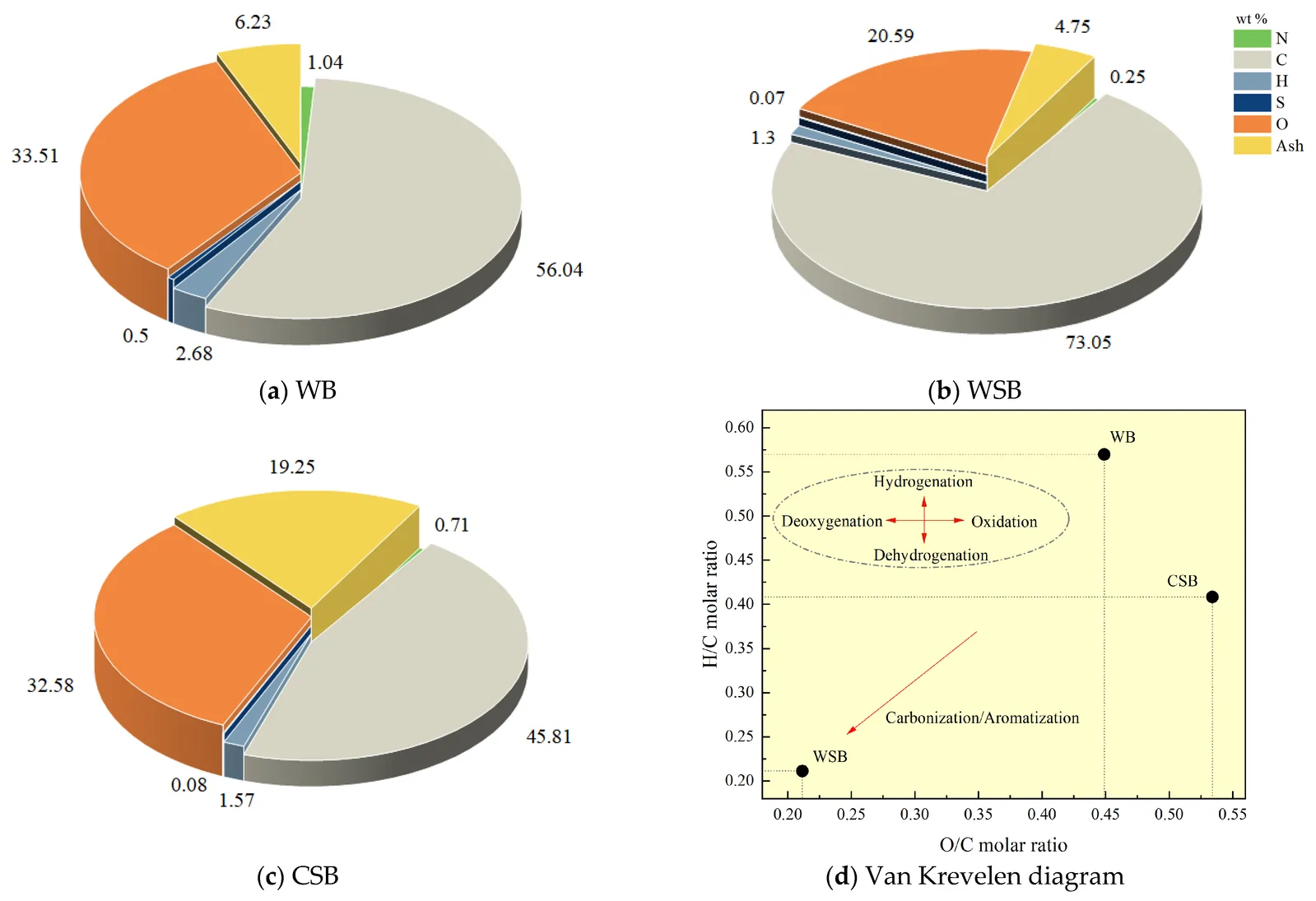
Elemental compositions and Van Krevelen diagram of biochars: (**a**–**c**) elemental compositions (wt.%); (**d**) Van Krevelen diagram.

As shown in Figure 4, the three biochars displayed distinct feedstock-dependent elemental characteristics. WSB had the highest carbon content and the lowest ash content, indicating stronger carbon enrichment and a lower inorganic contribution after pyrolysis. This may be associated with the lignin-rich and relatively mineral-poor characteristics of its walnut-shell precursor, consistent with the findings of Nair et al. [59]. WB showed a higher carbon content than CSB, while their oxygen contents were relatively similar. CSB exhibited the highest ash content among the three biochars, indicating a greater contribution of inorganic mineral residues in corn-straw biochar, consistent with the findings reported in reference [56]. In the Van Krevelen diagram, WSB showed the lowest H/C and O/C molar ratios, reflecting the highest degree of carbonization and aromatic condensation [42]. By contrast, the higher H/C ratio of WB suggested relatively lower aromatic condensation and greater retention of aliphatic moieties, whereas the higher O/C ratio of CSB reflected a greater contribution of oxygen-containing structures.

### 3.3. Functional Groups of Biochars

**Figure 5:**
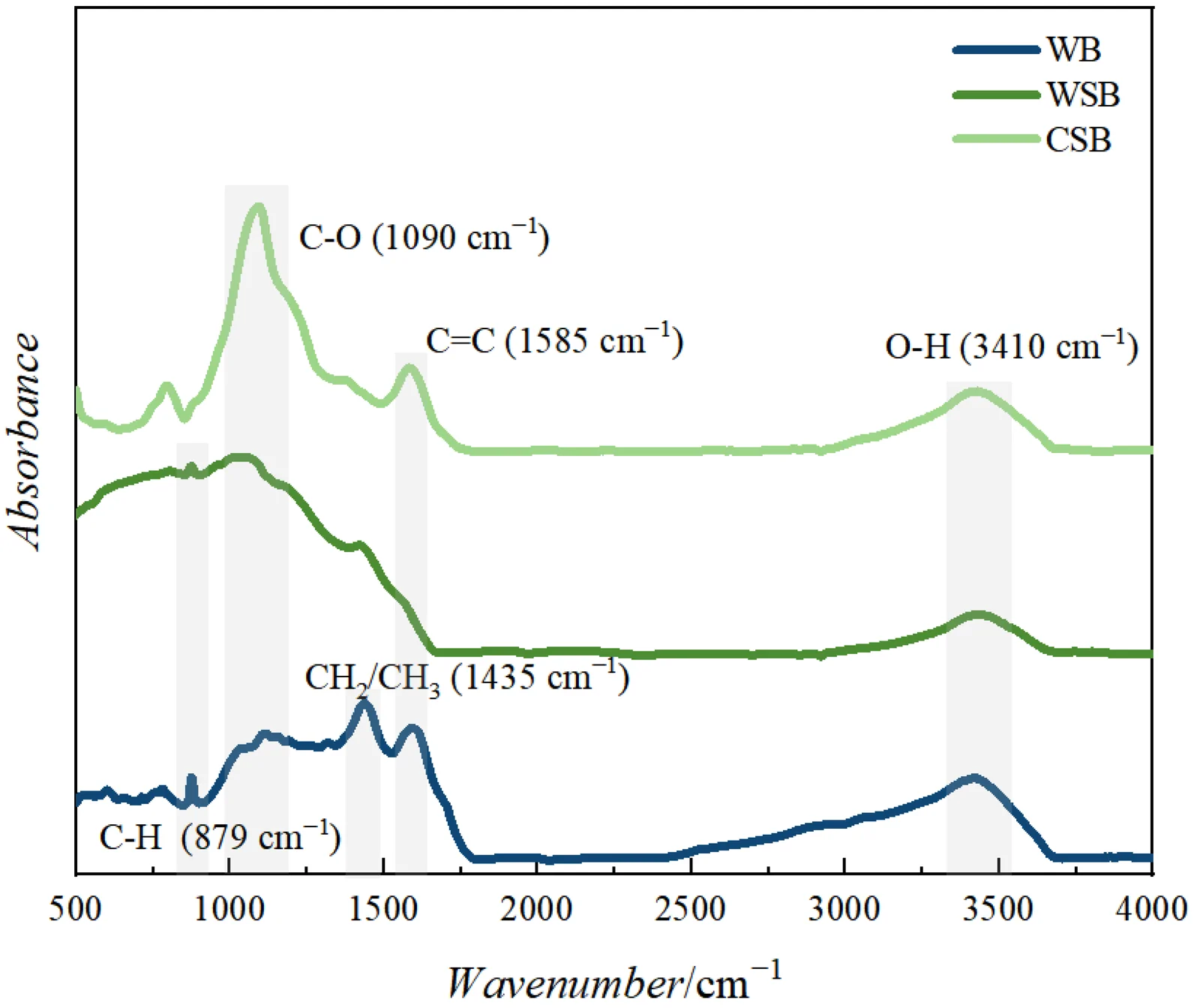
FTIR spectra of biochars.

Figure 5 reveals distinct feedstock-dependent FTIR characteristics among the three biochars. The absorption peak near 3410 cm^−1^ was associated with O-H stretching vibrations, while the band around 1585 cm^−1^ was mainly attributed to aromatic C=C skeletal stretching vibrations. CSB exhibited a more pronounced absorption band near 1090 cm^−1^, corresponding to C-O stretching vibrations, suggesting a greater contribution from oxygen-containing functional groups, consistent with its higher O/C ratio. WB showed a more pronounced band near 1435 cm^−1^, associated with aliphatic C-H deformation vibrations of CH_2_/CH_3_ groups. This observation is consistent with the higher H/C ratio of WB discussed in Section 3.2, suggesting greater retention of aliphatic functionalities and a comparatively lower degree of aromatic condensation [60]. In contrast, WSB exhibited weaker oxygen-related absorption features, particularly near 1090 cm^−1^, indicating a more deoxygenated carbon surface. The band near 879 cm^−1^, assigned to aromatic C-H out-of-plane bending vibrations, further indicated the presence of aromatic structures, particularly in WB and WSB. Overall, these feedstock-dependent differences suggest that CSB retains more polar surface functionalities, whereas WSB possesses a less oxygenated carbon surface that may favor hydrophobic adsorption.

### 3.4. Surface Chemical Properties of Biochars

**Figure 6:**
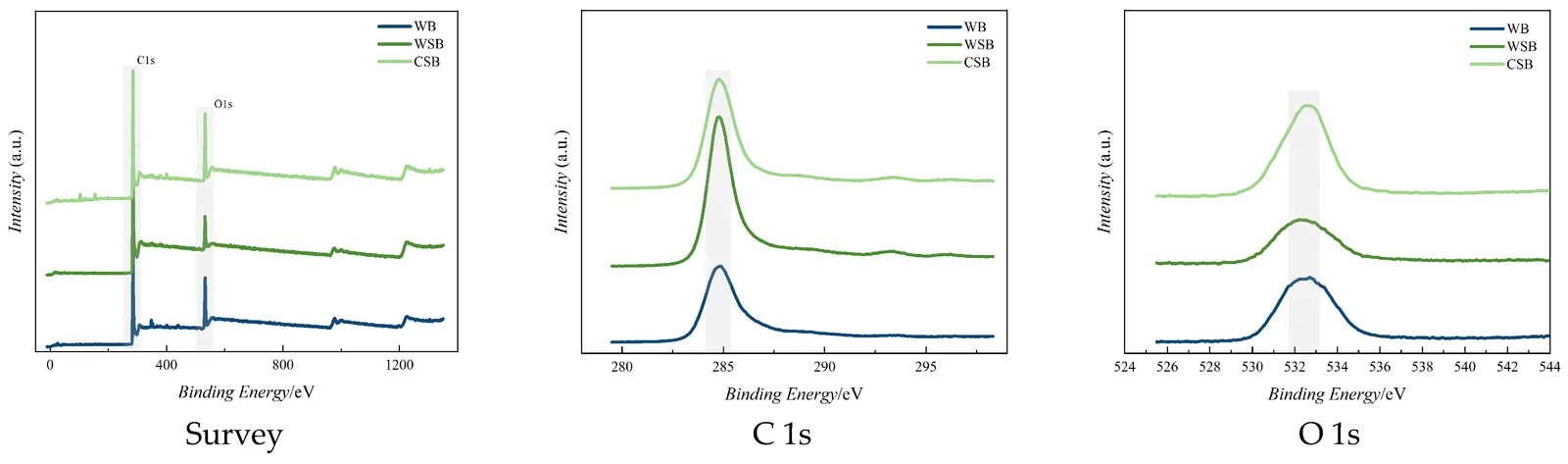
XPS survey and C 1s/O 1s spectra of biochars.

**Figure 7:**
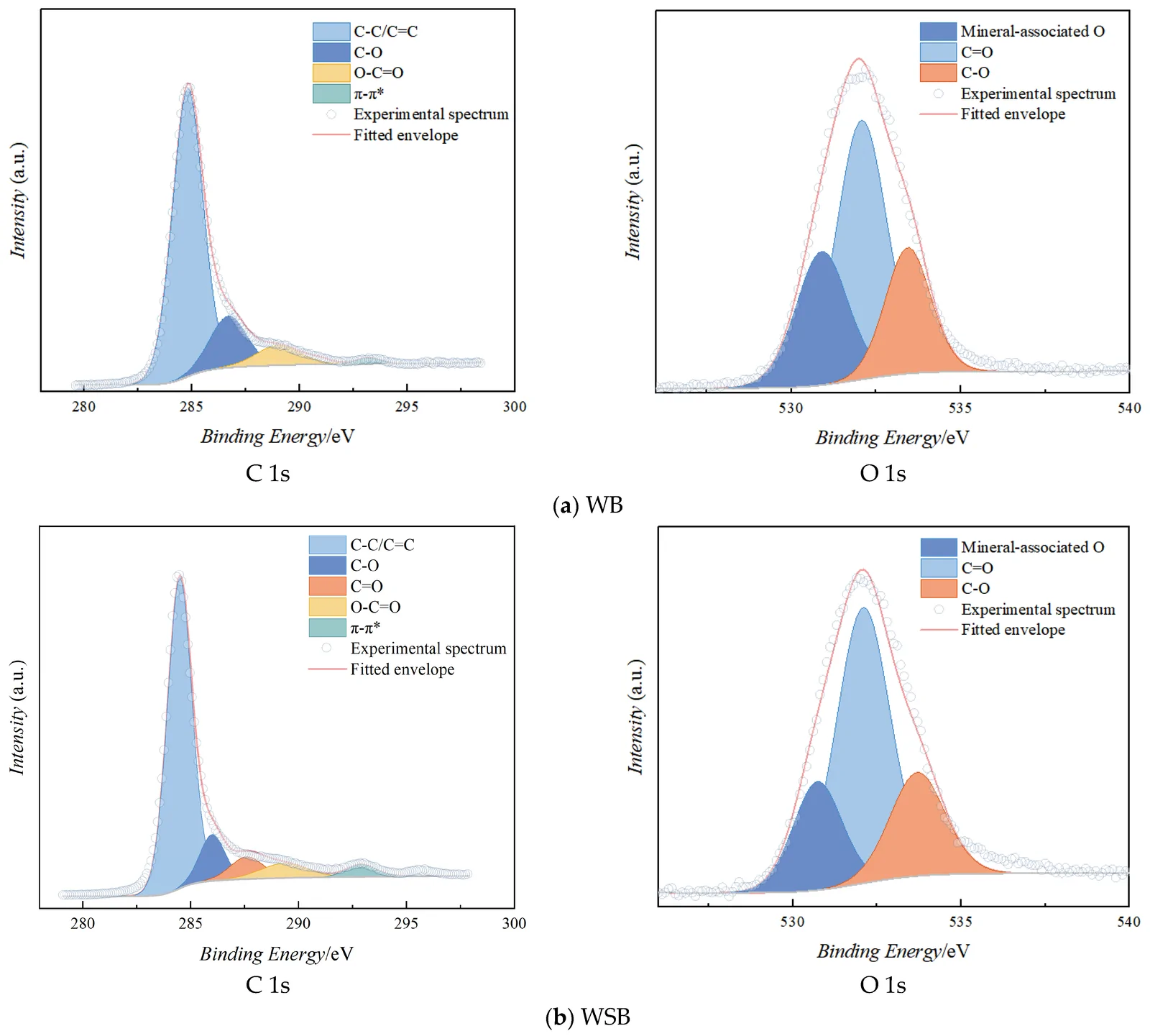

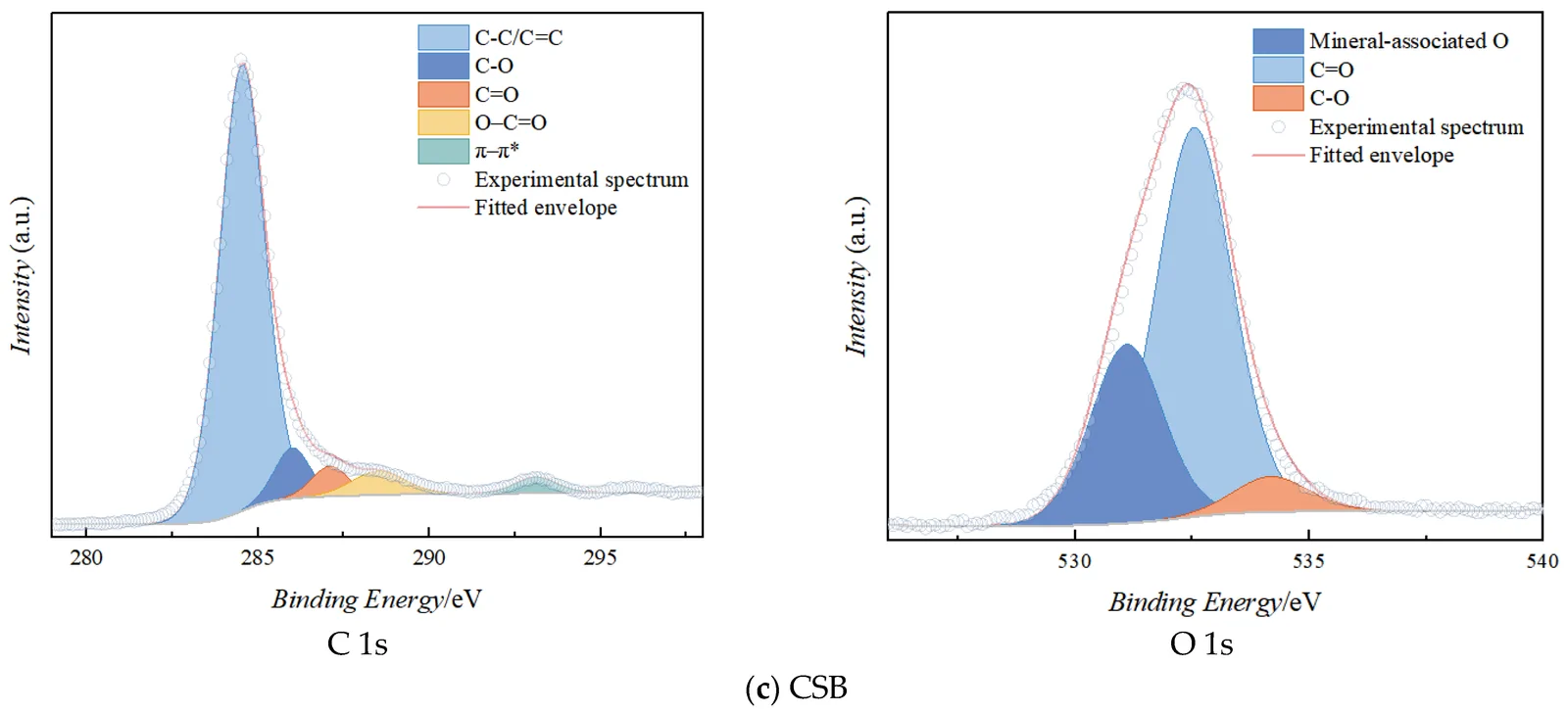
C 1s and O 1s high-resolution spectra of biochars.

As shown in Figure 6 and Figure 7, XPS survey spectra confirmed that C and O were the dominant surface elements of these three biochars, consistent with the elemental analysis. The high-resolution C 1s spectra were fitted with C-C/C=C, C-O, C=O and O-C=O components [61], together with a weak π-π* satellite at approximately 293 eV. The C-C/C=C component represents the carbon skeleton of the biochars, while C-O, C=O and O-C=O reflect oxygen-containing surface functionalities. WSB showed a relatively stronger carbon skeleton contribution, suggesting a more carbonized surface. In addition, the high-binding-energy satellite, which can be assigned to π-π* shake-up transitions associated with aromatic carbon domains, was more pronounced for WSB and CSB. The high-resolution O 1s spectra were resolved into three principal components. The low-binding-energy component at approximately 531 eV was tentatively assigned to mineral-associated oxygen species [62], while the components at approximately 532 eV and 533 eV were attributed to C=O and C–O species, respectively. CSB showed a greater relative contribution of mineral-associated oxygen than WB and WSB, consistent with its higher ash content and greater contribution of inorganic constituents.

### 3.5. Pore Characteristics of Biochars

**Figure 8:**
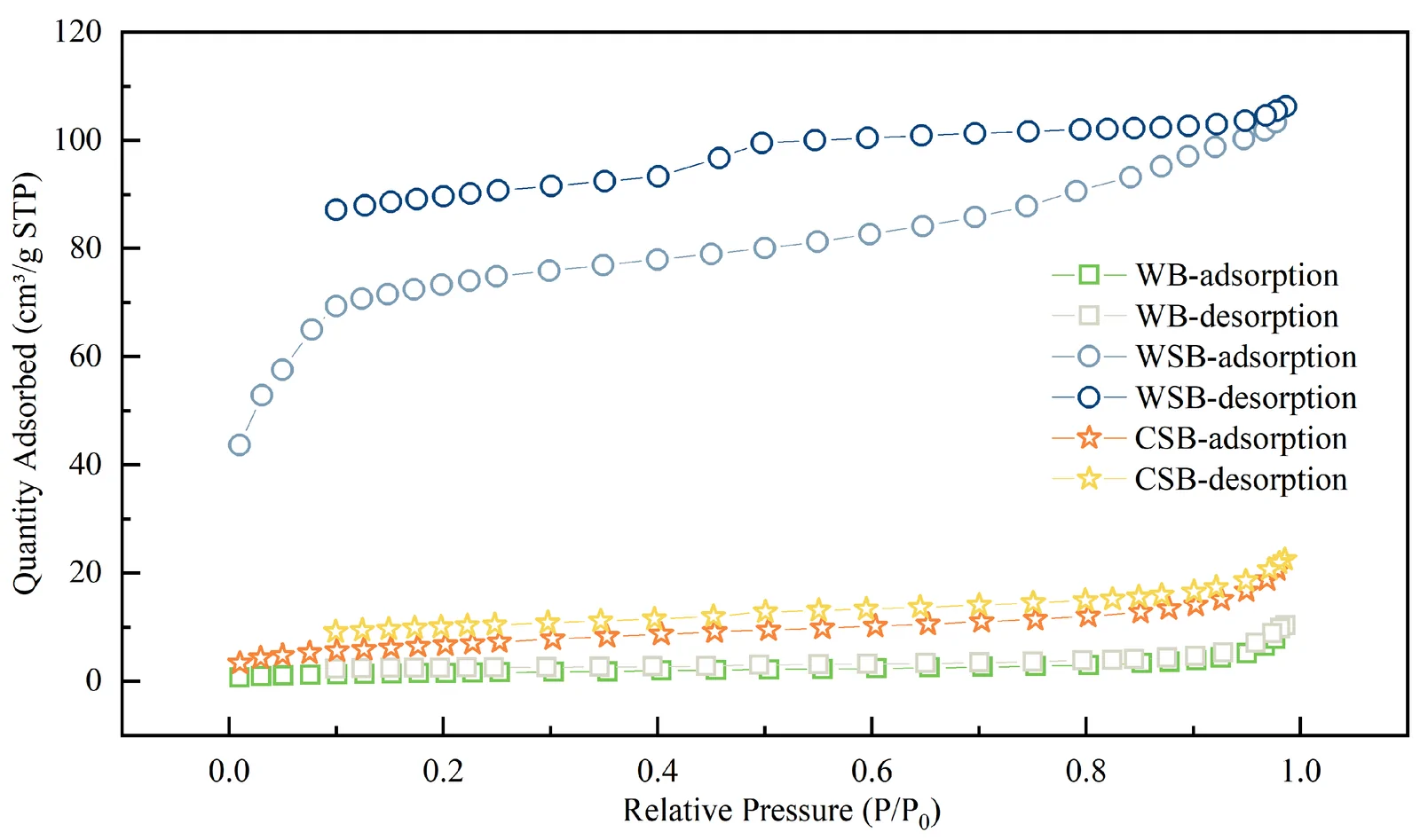
N_2_ adsorption–desorption isotherms of biochars.

As shown in Figure 8, N_2_ adsorption–desorption isotherms revealed clear feedstock-dependent differences in pore structure. WSB exhibited the highest N_2_ uptake, with a sharp increase at low relative pressure and a broad hysteresis loop between the adsorption and desorption branches, indicating the coexistence of abundant micropores and accessible mesopores. At *P*/*P*_0_ ≈ 0.01, the N_2_ uptake of WSB reached 43.62 cm^3^/g STP, much higher than that of CSB and WB, further suggesting its well-developed microporosity. CSB showed moderate adsorption with a gradual increase at high relative pressure, suggesting limited microporosity together with larger pores or intergranular voids. In contrast, WB displayed very low N_2_ adsorption over most of the pressure range, with only a slight increase near saturation, indicating poorly developed intrinsic porosity. This observation is consistent with previous studies showing that biochar pore architecture is strongly affected by biomass feedstock [42,44]. Overall, the N_2_ uptake and pore development followed the order WSB > CSB > WB.

**Figure 9:**
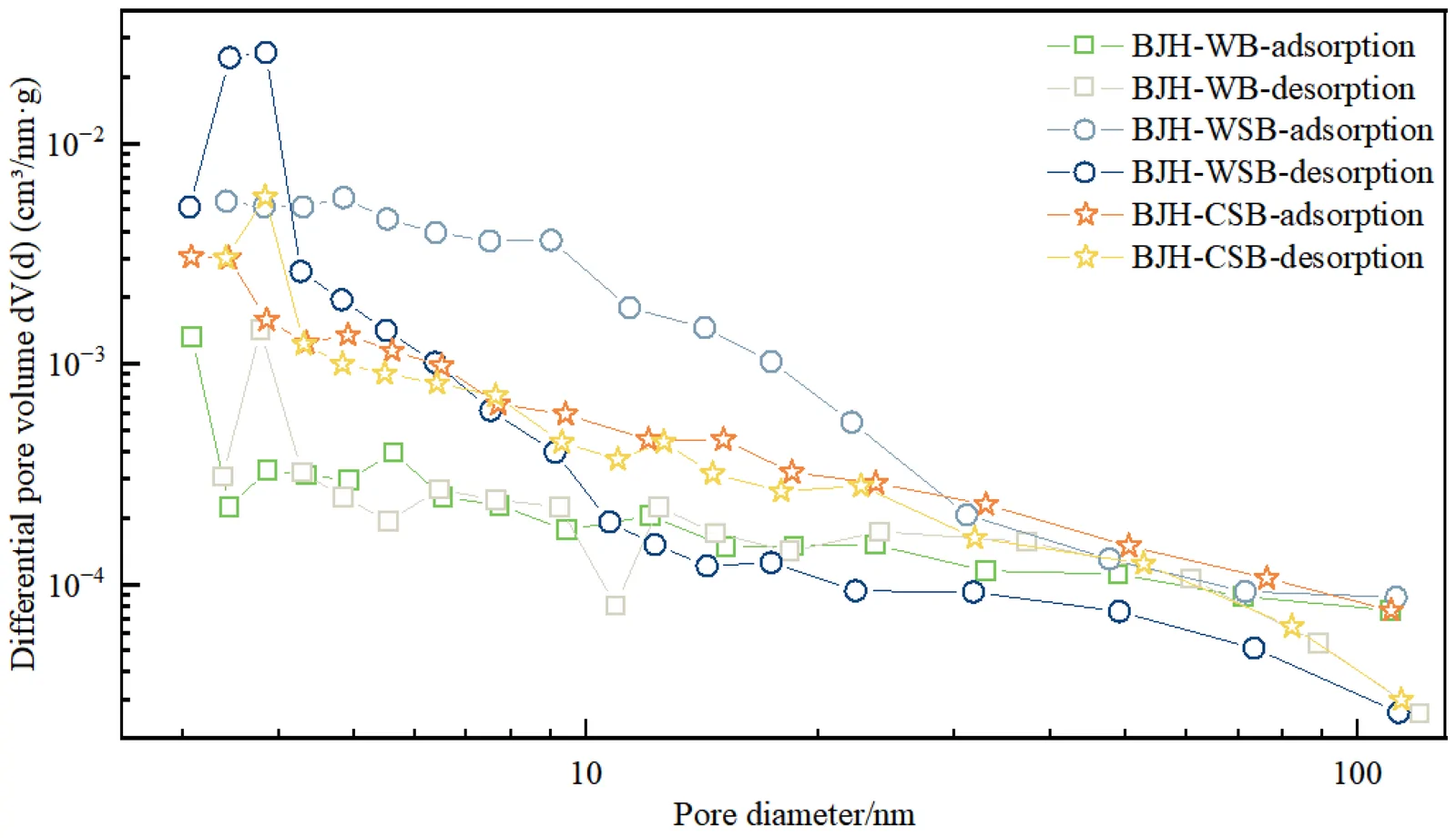
BJH pore size distributions of biochars.

The BJH pore size distributions shown in Figure 9 further illustrated pronounced differences in mesopore structure among the three biochars. WSB exhibited the highest differential pore volume in the small mesopore region, with the dominant pore sizes in the range of a few nanometers, indicating a well-developed mesoporous network. These small mesopores may provide accessible diffusion channels and retention sites for volatile and semi-volatile organic fume species. CSB showed a weaker but still distinguishable mesopore distribution, suggesting the presence of limited mesoporous channels together with larger transport pores. In contrast, WB displayed the lowest mesopore intensity, indicating poorly developed mesoporosity and potentially limited physical adsorption capacity.

As shown in Table 6, quantitative pore structure parameters further verified the pronounced differences among biochars derived from different feedstocks. WSB exhibited the largest specific surface area (285.8 m^2^/g), total pore volume (0.165 cm^3^/g), micropore volume fraction (56.5%) and external surface area (48.7 m^2^/g), confirming its well-developed hierarchical pore architecture. The dominant micropores may provide abundant adsorption sites for small volatile molecules. Representative VOCs, such as n-hexane, n-octane, benzene and toluene, have reported kinetic diameters of approximately 0.45–0.59 nm and may enter micropores with sufficiently large accessible openings [63]. Meanwhile, the considerable external surface area associated with mesopores enhanced mass transfer accessibility for fume components. CSB ranked second in specific surface area and total pore volume, with a higher proportion of 3–10 nm mesopores than WSB, indicating the presence of larger transport pores or open channels. By contrast, WB presented the lowest surface area, pore volume and micropore fraction, indicating poorly developed intrinsic porosity. The highest BET *C* constant of WSB further suggested stronger interaction between N_2_ molecules and the biochar surface, indicating its potentially favorable adsorption characteristics.

### 3.6. Real-Time Fume Suppression Behavior

**Figure 10:**
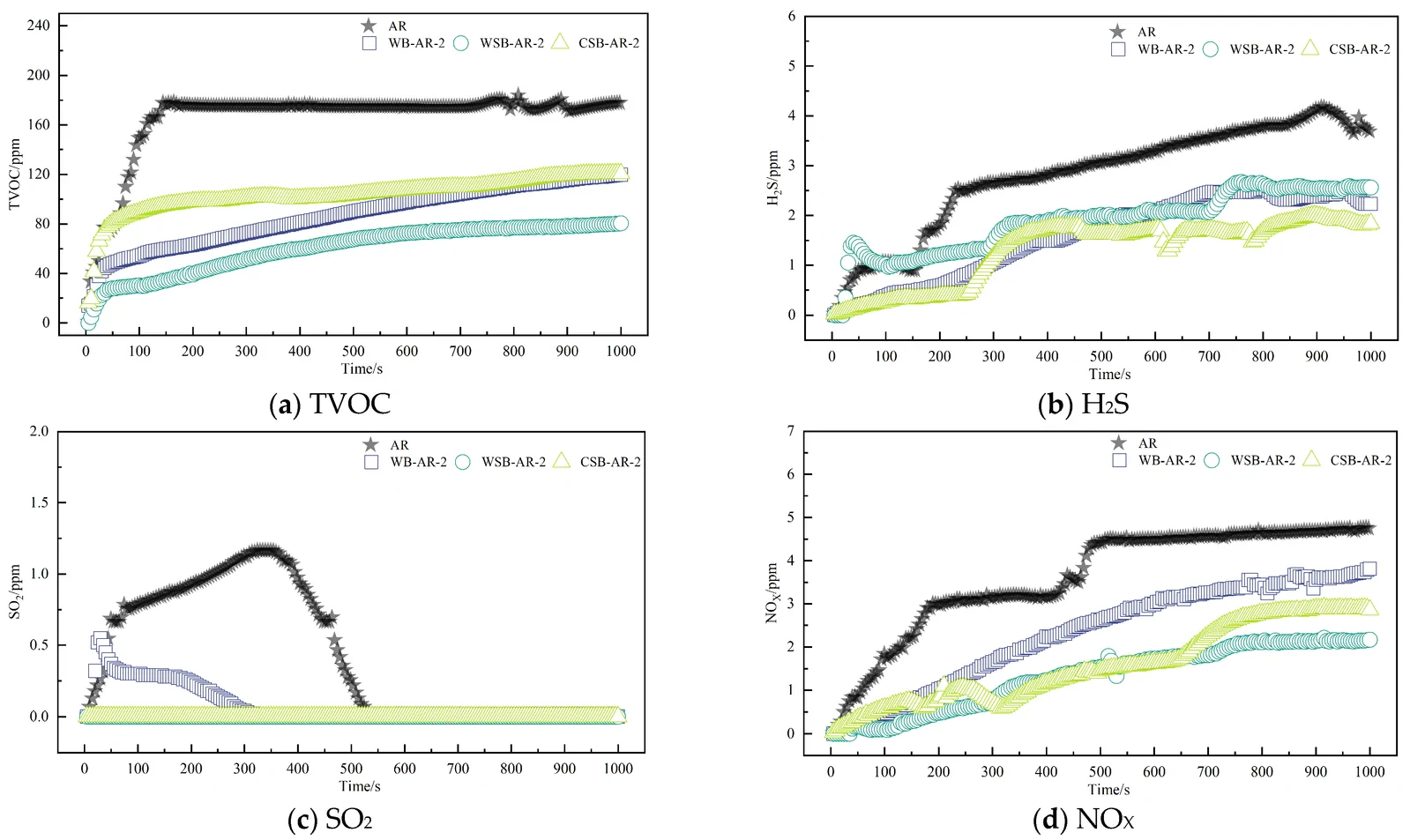
Real-time concentration profiles of pollutants from AR and biochar-modified AR.

As shown in Figure 10, biochar addition markedly reduced the real-time emissions of TVOC, H_2_S, SO_2_ and NO_X_ from AR binders. However, the suppression behavior was pollutant-dependent. WSB showed the strongest TVOC reduction, which was consistent with its high specific surface area, abundant micropores and hierarchical pore structure, suggesting that these characteristics may facilitate the physical adsorption and retention of volatile organic species. In addition to pore-mediated adsorption, biochar particles dispersed within the AR matrix may restrict the mobility and volatilization of asphalt constituents, contributing to the overall suppression effect [64,65]. For acidic sulfur gases, CSB exhibited the lowest H_2_S concentration, while both WSB and CSB maintained SO_2_ concentrations close to zero, indicating that acid gas suppression may be partly related to surface chemistry and mineral-associated sites. In particular, the relatively high ash content and oxygen-containing surface groups of CSB may provide additional polar or weakly alkaline adsorption sites, thereby contributing to the retention of sulfur-containing gases. For NO_X_, WSB and CSB generally exhibited stronger suppression than WB, which may be associated with differences in pore architecture and surface characteristics among the three biochars.

**Figure 11:**
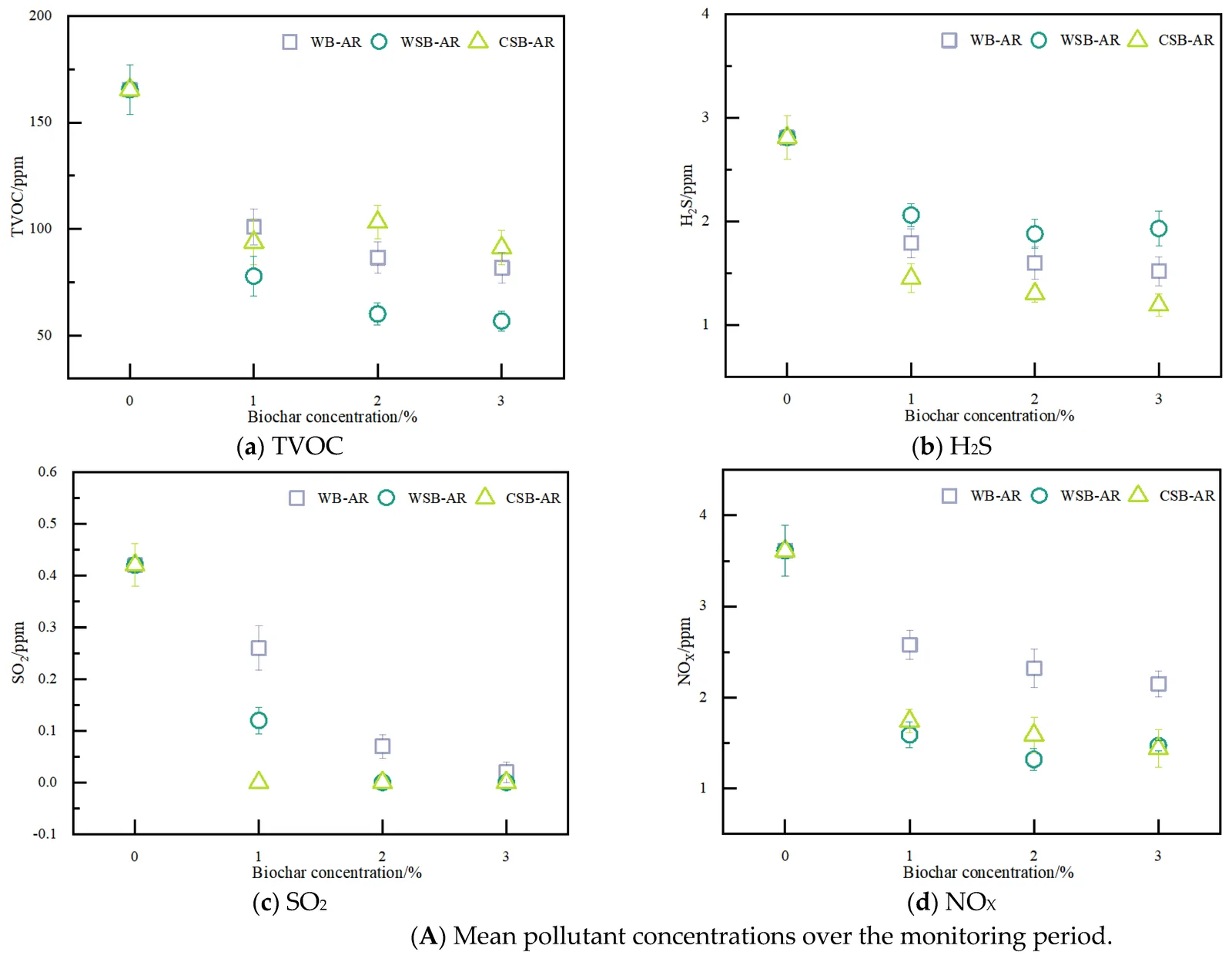

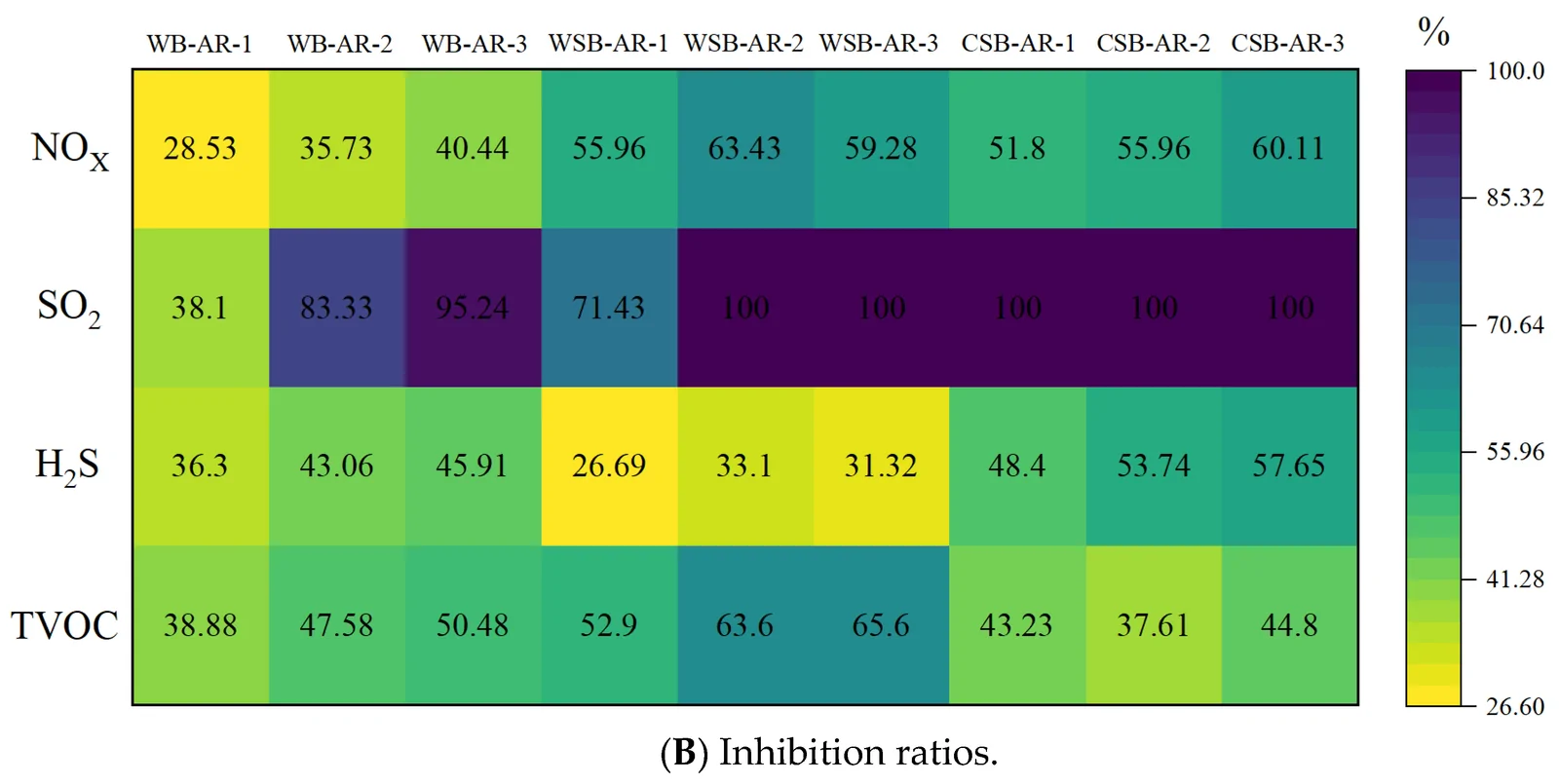
Mean concentrations and inhibition ratios of pollutants at different biochar dosages.

Figure 11 compares the mean pollutant concentrations and inhibition ratios over the 1000 s monitoring period. For TVOC, WSB showed the strongest inhibition, with the inhibition ratio increasing from 52.90% to 65.60% as the dosage increased from 1 to 3 wt%, consistent with its developed hierarchical pore structure and potentially greater physical adsorption capacity for organic species. WB showed a moderate improvement with increasing dosage, whereas CSB exhibited weaker TVOC suppression. For H_2_S, CSB outperformed the other two biochars. Its H_2_S inhibition increased from 48.40% to 57.65%, which may be associated with its relatively higher ash content and more abundant oxygen-containing surface groups. For SO_2_, CSB completely suppressed the emission across the tested dosage range, while WSB reduced SO_2_ to zero at 2 and 3 wt%. For NO_X_, WSB and CSB were both effective, with WSB showing the lowest concentration at 2 wt%, whereas WB remained the least effective. As shown in Table 7, both biochar type and dosage significantly affected the inhibition ratios of all four pollutants (all *p* < 0.05), further supporting the observed suppression patterns. However, the improvement in pollutant reduction became less pronounced when the dosage increased from 2 to 3 wt%, indicating a smaller marginal gain at higher biochar dosages. These results support the selection of 2 wt% as a representative intermediate dosage for further TD-GC-MS and DSR analyses, allowing the effects of different biochar feedstocks to be compared under identical addition conditions.

### 3.7. TD-GC-MS Composition of Organic Fume Species

**Figure 12:**
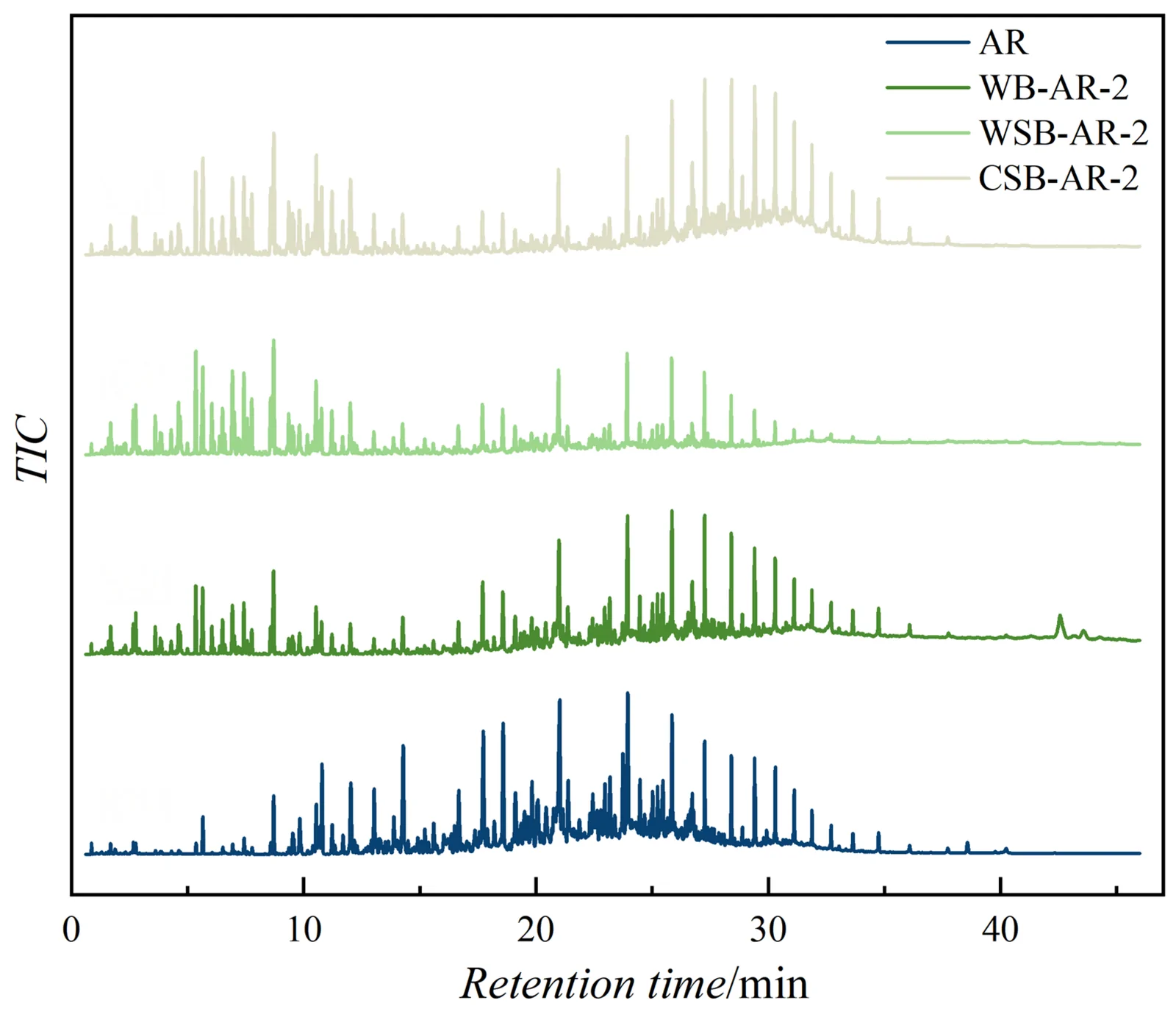
TIC chromatograms of AR and biochar-modified AR fumes.

Figure 12 and Table 8 summarize the overall TD-GC-MS profiles of AR and biochar-modified AR fumes. Biochar incorporation clearly altered both the number of detectable peaks and their retention-time distribution. Compared with AR fume, all biochar-modified samples showed fewer valid peaks and unique tentative assignments, indicating a reduction in the chemical complexity of detectable organic fumes. WSB-AR-2 exhibited the fewest peaks, suggesting a simpler detectable organic-fume profile under the analytical conditions used. The TIC profiles further showed that biochar addition did not produce a uniform decrease in peak signals, but rather a feedstock-dependent redistribution of peak signals across the whole chromatographic window. Notably, the biochar-modified samples exhibited more peaks in the 0–10 min retention-time region. This may reflect a redistribution of the residual organic-fume composition toward more early-eluting species after biochar addition. WB-AR-2 showed moderate suppression, whereas CSB-AR-2 retained a relatively complex chromatographic profile, especially in the middle-to-late retention-time region. The pronounced reduction in detectable organic species for WSB-AR-2 is consistent with its developed pore architecture, suggesting that pore-mediated physical adsorption and retention may contribute to the selective suppression of organic fumes.

**Figure 13:**
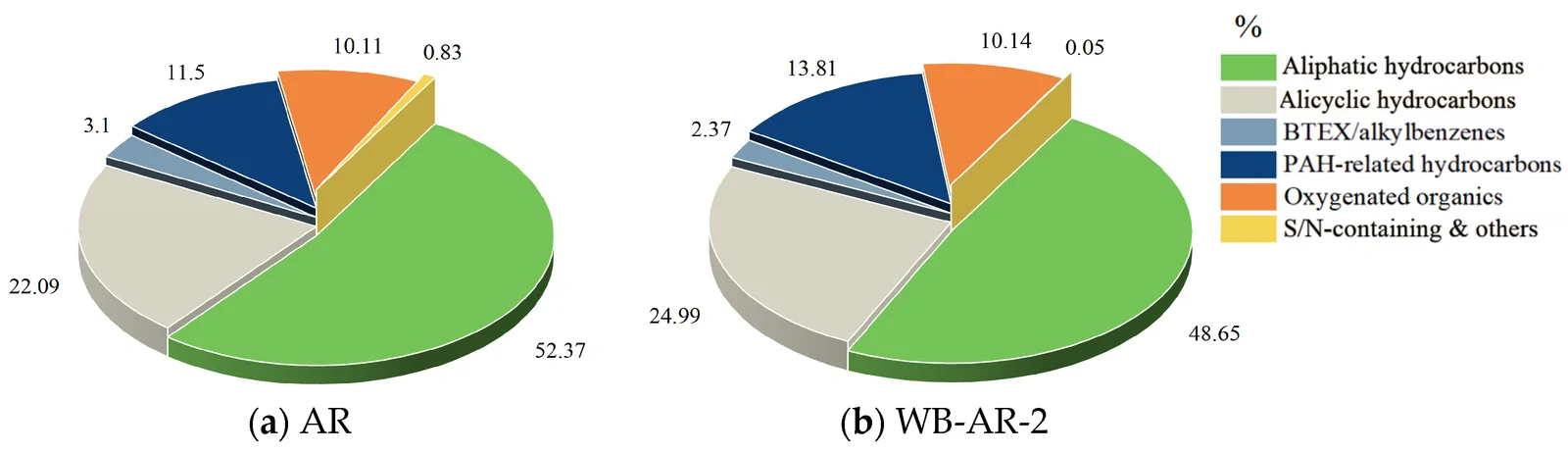

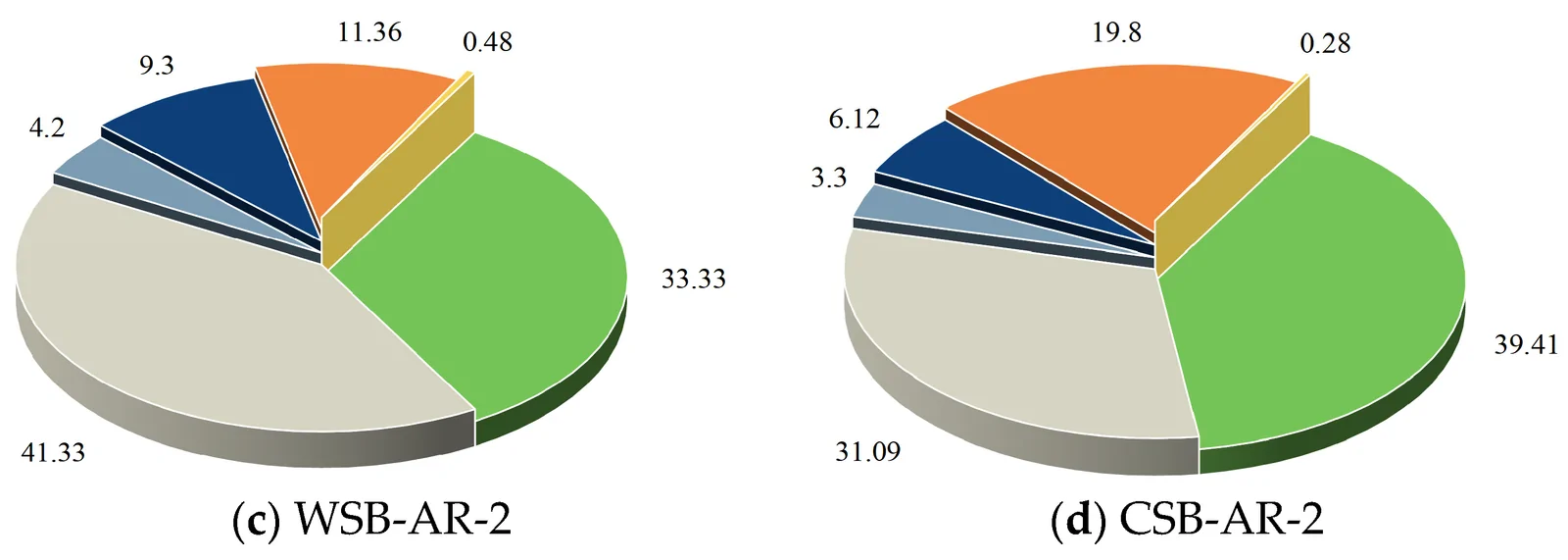
Chemical-category distributions of AR and biochar-modified AR fume.

Figure 13 illustrates the chemical-category distributions of AR and biochar-modified AR fumes. AR fumes were mainly composed of aliphatic hydrocarbons, followed by alicyclic hydrocarbons, PAH-related hydrocarbons and oxygenated organics, while BTEX/alkylbenzenes and S/N-containing compounds accounted for smaller fractions. After biochar addition, the category distribution varied with biochar feedstock. Fumes from WB-AR-2 retained a composition similar to that of AR fumes, with smaller fractions of BTEX/alkylbenzenes and S/N-containing compounds. WSB-AR-2 exhibited a markedly decreased proportion of aliphatic hydrocarbons but a higher alicyclic hydrocarbon contribution, indicating selective compositional redistribution. CSB-AR-2 showed the highest oxygenated organic fraction and a reduced PAH-related contribution. Nevertheless, the compositional redistribution should not be interpreted as an overall environmental benefit. These results demonstrate that biochar addition induces feedstock-specific changes in the relative organic-fume composition, in line with the differences in pore structure and surface chemistry among the three biochars.

**Figure 14:**
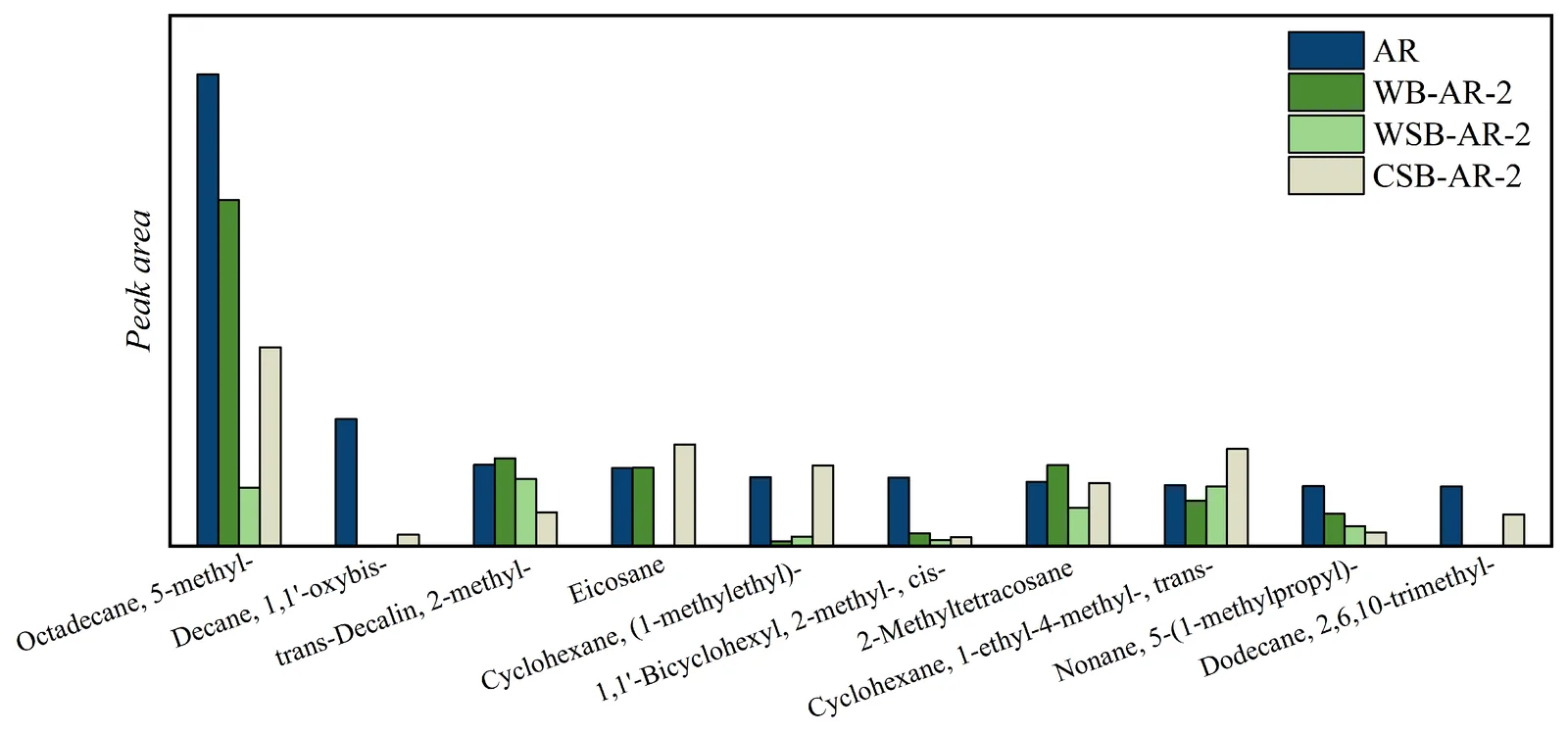
Influence of biochars on Top 10 compounds in AR binder fume.

Figure 14 illustrates the changes in the Top 10 compounds in AR binder fume after the addition of biochars. Biochar addition reduced most of the dominant AR components, with WSB showing the most pronounced attenuation, particularly for Octadecane, 5-methyl-, Decane, 1,1′-oxybis-, Dodecane, and 2,6,10-trimethyl-. WB showed moderate suppression for most species. By contrast, several high-carbon or cyclic assignments showed increased residual responses in specific samples, especially CSB-AR-2, indicating selective compositional redistribution.

**Figure 15:**
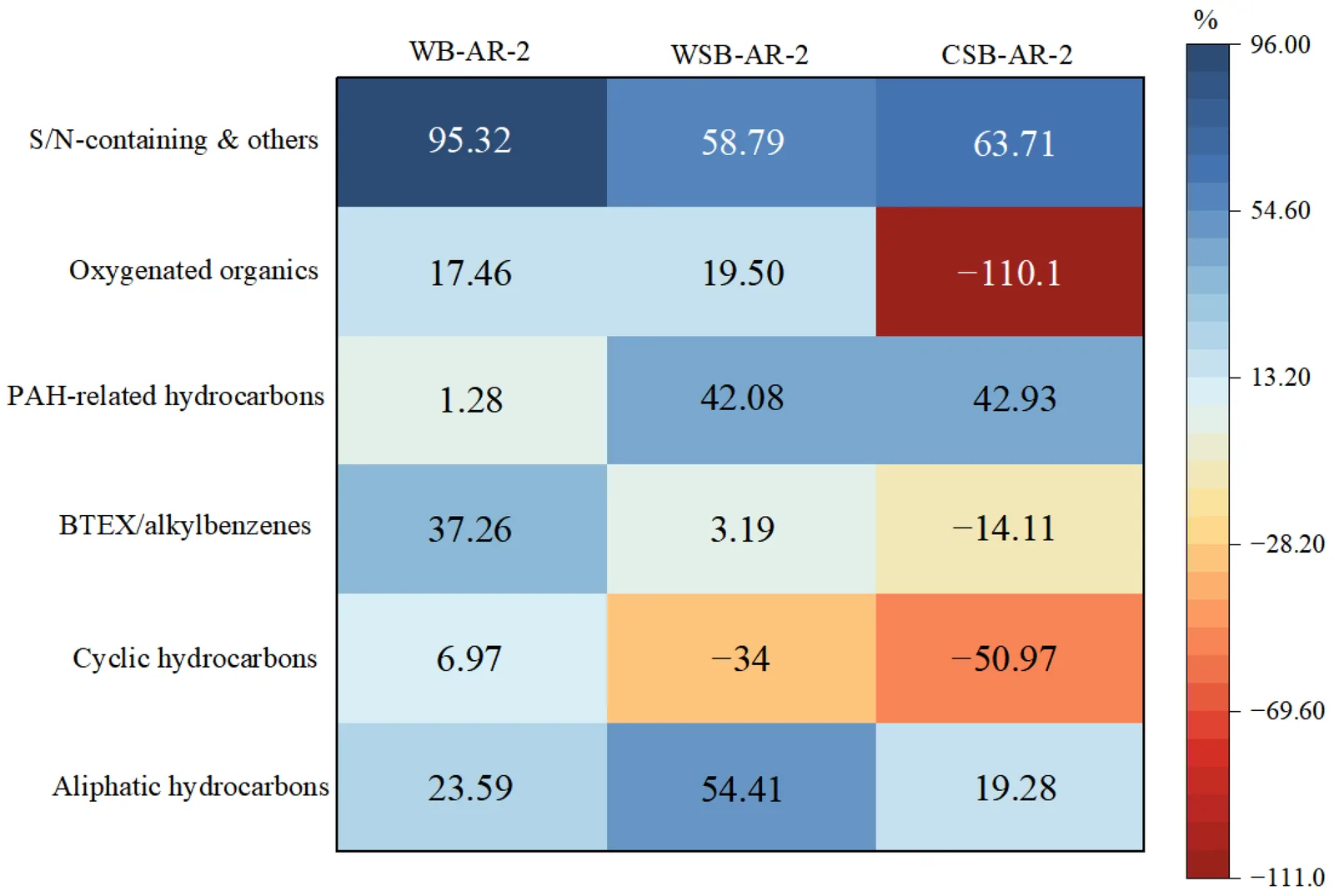
Category-level TD-GC-MS inhibition ratios after biochar modification. Note: Negative inhibition ratios indicate increased relative responses compared with the AR binder. These values do not quantify changes in absolute pollutant concentrations or emitted masses.

Figure 15 further shows that the semi-quantitative inhibition ratios, calculated from relative TIC peak areas, are strongly category-dependent. WSB showed strong reductions in the relative responses of aliphatic hydrocarbons and PAH-related hydrocarbons, consistent with its developed microporosity and accessible mesoporous structure, which may facilitate physical adsorption and retention of organic species with different molecular sizes. WB exhibited the most balanced suppression pattern across all six categories and was particularly effective for BTEX/alkylbenzenes and S/N-containing compounds. In contrast, CSB reduced PAH-related and S/N-containing responses but showed increased responses for alicyclic hydrocarbons and oxygenated organic compounds, suggesting that its oxygen-rich and mineral-containing surface may alter the retention, release or secondary transformation pathways of organic fragments during AR heating. These results suggest that the selective suppression of organic fumes may arise from the combined effects of pore-mediated physical retention and surface chemical interactions, with their relative contributions depending on biochar feedstock. Nevertheless, the semi-quantitative TD-GC-MS results are insufficient to establish an overall reduction in toxicity or health risk.

### 3.8. Binder Properties and Rheological Behavior

**Figure 16:**
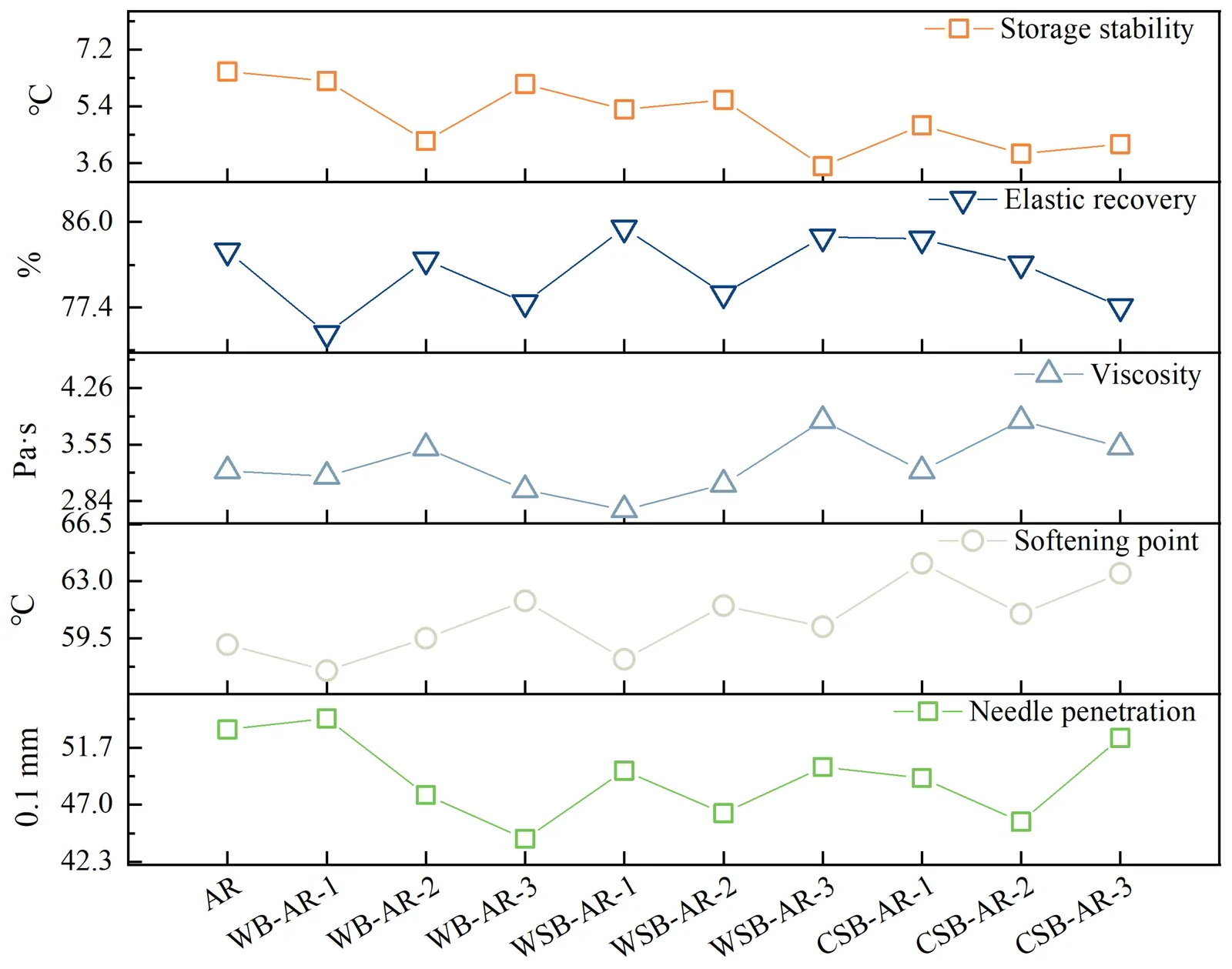
Conventional properties of AR and biochar-modified AR binders.

As shown in Figure 16, compared with pristine AR binder, the majority of biochar-modified AR samples exhibited lower needle penetration values and higher softening points, corresponding to enhanced binder stiffness and improved high-temperature deformation resistance. This behavior may be associated with the reinforcing effect of biochar particles, interfacial interactions with asphalt constituents, and potential adsorption or retention of relatively mobile asphalt fractions. The rigid carbonaceous framework of biochar may restrict the mobility of asphalt molecules, while its porous structure may promote the adsorption or retention of relatively mobile asphalt fractions, potentially contributing to the increased stiffness of the continuous phase, consistent with previous studies [26,64,65]. The rotational viscosity generally increased slightly after biochar addition, indicating greater resistance to flow. The storage stability index was reduced for most biochar-modified AR binders, suggesting improved compatibility and reduced phase separation. This improvement may be associated with the increased binder viscosity and possible interfacial interactions between biochar surfaces, asphalt fractions and CR particles. In contrast, elastic recovery showed only limited variation among different biochar types and dosages, indicating that the recoverable elastic response remained primarily governed by the CR network rather than by biochar particles.

**Figure 17:**
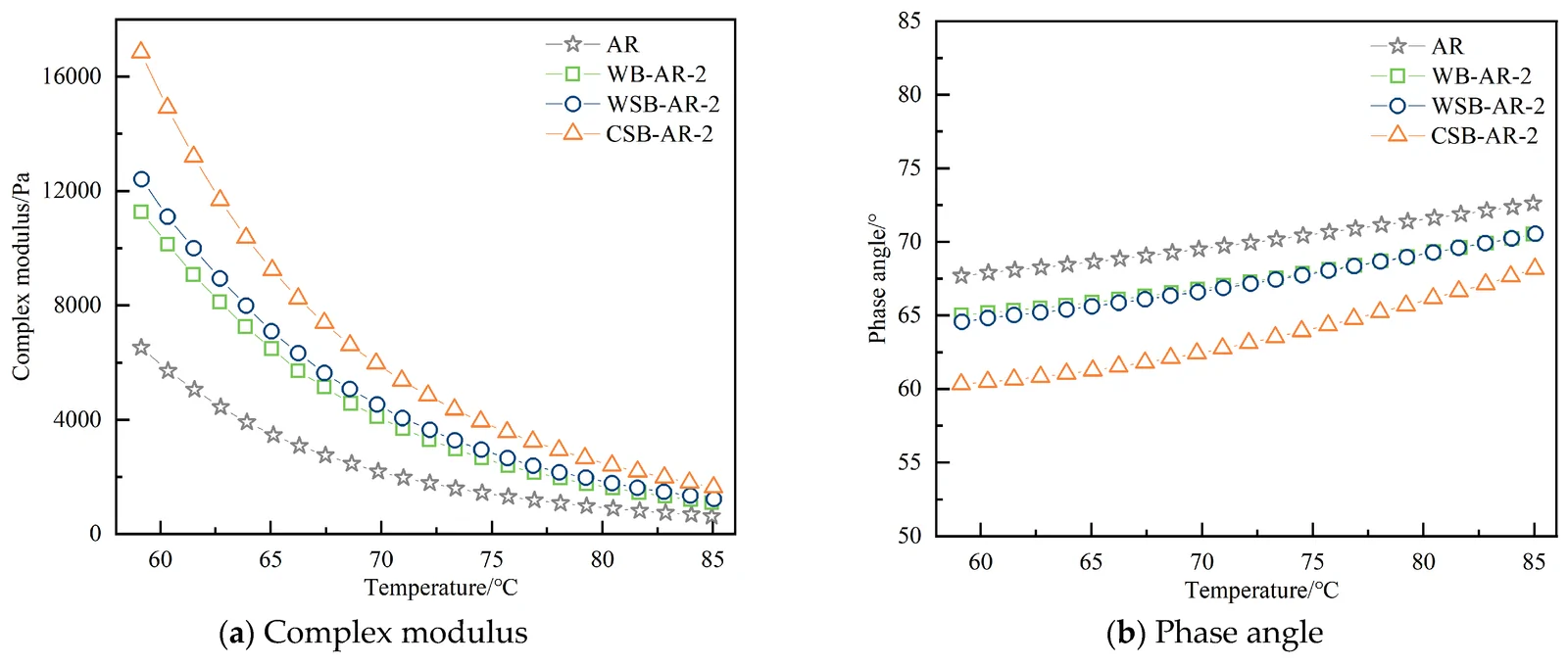
Dynamic rheological properties of AR and biochar-modified AR binders.

Figure 17 shows that all binders exhibited typical thermal softening, with decreasing complex modulus and increasing phase angle as temperature increased. Compared with AR binders, the biochar-modified binders showed higher complex modulus values and lower phase angles, indicating increased high-temperature stiffness, consistent with conventional tests. CSB exhibited the greatest increase in complex modulus and decrease in phase angle, whereas WSB and WB showed more moderate changes. This may be related to the higher ash content and richer oxygen-containing surface groups of CSB, which could enhance the filler effect and interfacial interactions with polar asphalt fractions and swollen CR particles, thereby restricting molecular mobility and improving the high-temperature viscoelastic response of binders.

### 3.9. Biochar Inhibition Mechanism

**Figure 18:**
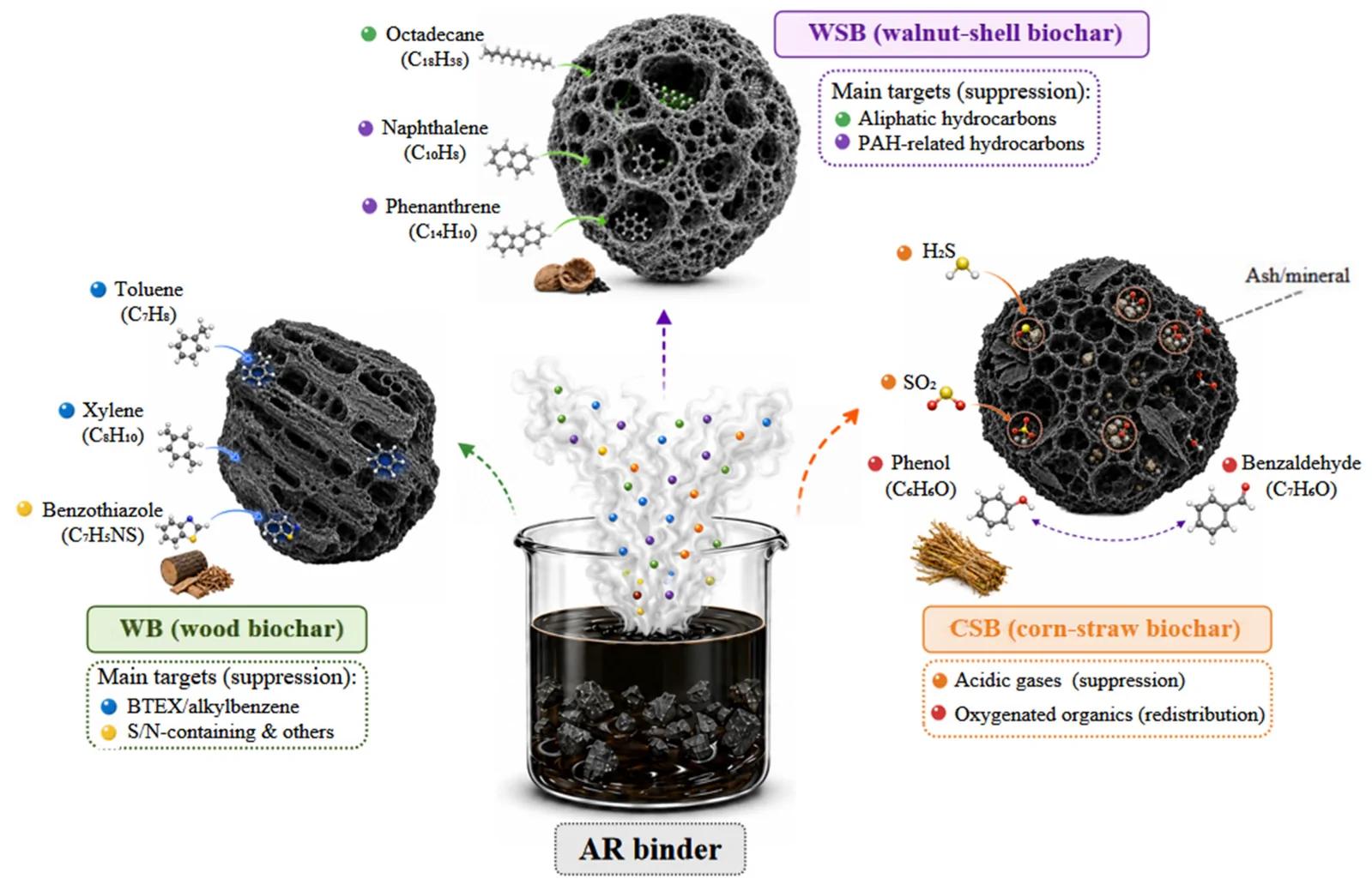
Proposed mechanisms of AR fume suppression by biochars.

As shown in Figure 18, biochar-mediated AR fume suppression is a feedstock-dependent and multi-pathway process. The superior TVOC suppression of WSB may be partly associated with its hierarchical pore network, high specific surface area and abundant micropores, which may provide favorable adsorption domains and diffusion pathways for organic fume species, particularly aliphatic hydrocarbons and PAH-related compounds. CSB, despite its less developed pore structure, exhibited higher ash content and more abundant oxygen-containing surface groups, which may contribute to the retention of acidic gases and the reduction in PAH-related responses. However, the increased semi-quantitative responses of oxygenated organic compounds suggest that CSB may also induce compositional redistribution of residual fumes rather than uniform suppression. Although WB had less developed porosity, it selectively reduced BTEX/alkylbenzenes and S/N-containing responses, potentially through interactions involving surface aromatic domains and functional groups. Overall, biochar-mediated AR fume suppression may involve the combined contributions of physical adsorption and surface chemical interactions. Micropores may favor pore filling by small VOC molecules, while mesopores may promote mass transfer and retention of relatively larger organic species. Aromatic carbon domains may contribute to the retention of aromatic organic species through hydrophobic and π–π interactions, whereas ash-derived minerals and oxygenated sites may contribute to acidic sulfur-gas retention. Additionally, biochar particles dispersed within the AR matrix may hinder the migration and volatilization of asphalt constituents, consistent with the increased rotational viscosity and complex modulus observed in biochar-modified binders. These coupled pathways vary with biomass feedstock, resulting in pollutant-specific suppression by WSB, WB and CSB. Nevertheless, the reported suppression efficiencies are specific to the tested AR formulation and controlled laboratory conditions, and their applicability to other formulations, processing temperatures and field-production conditions requires further validation.

## 4. Conclusions

This study evaluated three widely available biochars as fume-suppressing additives for AR binder and investigated their potential suppression mechanisms by combining material characterization, real-time emission monitoring, TD-GC-MS analysis and binder tests. The main conclusions are as follows:

(1) WSB exhibited the most developed hierarchical pore structure, with the highest BET surface area of 285.8 m^2^/g and the largest total pore volume of 0.165 cm^3^/g. Its abundant micropores and accessible mesopores may provide favorable adsorption sites and diffusion pathways for organic fume species. CSB showed moderate porosity with more prominent large transport pores, while WB exhibited the least developed intrinsic porosity, suggesting comparatively limited pore-mediated adsorption potential.
(2) WSB featured a highly carbonized, relatively deoxygenated carbon surface with low ash content. CSB contained abundant ash-derived minerals and oxygen-containing surface groups, potentially providing polar surface sites for pollutant retention. WB showed a less-condensed carbon structure, as indicated by its higher H/C ratio and more pronounced C–H absorption band.
(3) Real-time monitoring revealed pollutant-dependent emission reduction. WSB showed the most pronounced suppression of TVOC and, together with CSB, exhibited substantial NO_X_ inhibition. CSB exhibited better H_2_S inhibition, with an inhibition ratio of 57.65%, and achieved complete suppression of the SO_2_ response across the tested dosage range. The additional improvement was less pronounced when the dosage increased from 2 wt% to 3 wt%, indicating a weakened marginal suppression effect.
(4) TD-GC-MS results confirmed selective suppression and compositional redistribution. WSB-AR showed the fewest valid peaks, indicating the lowest detectable organic-fume complexity. Category-level analysis showed that WSB mainly reduced the relative responses of aliphatic and PAH-related hydrocarbons, WB selectively reduced BTEX/alkylbenzenes and S/N-containing compounds, while CSB reduced PAH-related and S/N-containing responses but showed increased responses for oxygenated organics and alicyclic hydrocarbons.
(5) The biochar-modified binders generally showed lower needle penetration values, higher softening points, increased viscosity and improved storage stability. Rheological results further confirmed increased high-temperature stiffness of the AR binder. Therefore, biochar dosage should be optimized by balancing fume suppression and high-temperature binder stiffness.

Overall, this study reveals the feedstock-dependent and pollutant-specific suppression behavior of biochars in AR fumes and verifies the feasibility of achieving emission reduction alongside improved high-temperature binder stiffness. These findings suggest that pore-mediated physical retention and surface chemical interactions may jointly contribute to the observed emission reductions. However, the semi-quantitative TD-GC-MS results cannot establish an overall reduction in toxicity or health risk. Future work may focus on validation under field-construction conditions, optimization of surface-modified biochars, and evaluation of the long-term aging performance of modified binders under different working conditions.

## Figures and Tables

**Table 1:** Main pollutants emitted from typical asphalt binders during construction.

Pollutant Category	Name	Base Asphalt	SBS Modified Asphalt	Asphalt Rubber	Reference
Inorganic Sulfur-Containing Gases	Sulfur dioxide	√	√	↑	[14,24]
	Hydrogen sulfide	√	√	↑	[6,14,25,26]
Oxygenated Organic Compounds	Acetaldehyde	√	√	√	[14,24]
	Acetone	√	√	√	[13,14]
	Octanal	—	—	√	[6]
	Decanal	—	—	√	[6]
	2,4,6-tri-tert-butylphenol	—	√	—	[15]
Aliphatic Hydrocarbons	n-alkanes C11, C12, C17, C20~C22, C24	√	√	√	[6,13,15,27,28]
Monocyclic Aromatic Hydrocarbons	Benzene	√	√	↑	[13,14,24]
	Toluene	√	√	↑	[8,13,24,29]
	Xylenes (m-/p-/o-)	√	√	↑	[8,13,24,29]
	1,2,4-trimethylbenzene	√	√	√	[13,24]
	p-diethylbenzene	√	√	√	[24]
Polycyclic Aromatic Hydrocarbons	Naphthalene	√	√	↑	[6,8,13,14,29]
	Fluorene	√	√	↑	[6,15]
	Phenanthrene	√	√	↑	[6,15]
	Anthracene	○	○	√	[6]
Heterocyclic Aromatic Compounds	Benzothiazole	—	—	√	[6,8,15,25]
	4-methyldibenzothiophene	—	—	√	[6]
	2,2,4-trimethyl-1,2-dihydroquinoline	—	—	√	[6,8]

Note: √: detected in asphalt binder; ↑: more prominent in AR binder; ○: detected but at low levels or inconsistently; —: not detected or not reported.

**Table 2:** Properties of base asphalt.

Physical Properties	Unit	Requirements	Test Results	Test Methods
25 °C needle penetration	0.1 mm	60–80	71	T 0604
Penetration index	/	−1.5~+1.0	−1.1	T 0604
Softening point	°C	≥46	48.0	T 0606
Ductility at 10 °C	cm	≥15	33	T 0605
Ductility at 15 °C	cm	≥100	>150	T 0605
Solubility	%	≥99.5	99.96	T 0607
Flash-point	°C	≥260	>300	T 0611

**Table 3:** Properties of crumb rubber.

Item	Unit	Test Results	Test Methods
Heating loss	%	0.62	GB/T 19208-2008 [48]
Ash	%	8.75	
Iron content	%	0.029	
Fiber content	%	0	
Residue on sieve	%	0.014	

**Table 4:** Binder samples.

Sample Abbreviations	Composition	Purpose
AR	Base asphalt + 20 wt% CR	Control
WB-AR-1/2/3	AR + 1/2/3 wt% WB	Real-time monitoring and binder tests
WSB-AR-1/2/3	AR + 1/2/3 wt% WSB	Real-time monitoring and binder tests
CSB-AR-1/2/3	AR + 1/2/3 wt% CSB	Real-time monitoring and binder tests
WB-AR-2/WSB-AR-2/CSB-AR-2	AR + 2 wt% biochar	TD-GC-MS analysis, DSR tests

**Table 5:** Detection specifications of the gas analyzer.

Analyzer	Sensor	Range	Resolution
TVOC	PID photoionization	0–500 ppm	0.1 ppm
H_2_S	Electrochemical	0–100 ppm	0.01 ppm
SO_2_	Electrochemical	0–100 ppm	0.01 ppm
NO_X_	Electrochemical	0–100 ppm	0.01 ppm

**Table 6:** Pore-structure parameters of WB, WSB, and CSB derived from N_2_ physisorption analysis.

Parameter	WB	WSB	CSB
Specific surface area (m^2^/g)	5.5	285.8	24.5
Total pore volume (cm^3^/g)	0.0160	0.165	0.035
Micropore volume percentage (%)	2.29	56.50	7.35
Mesopore (3–10 nm) surface-area contribution (%)	70.8	18.7	32.6
External surface area (m^2^/g)	4.5	48.7	18.7
BET constant *C*	70.8	130.6	73.0

**Table 7:** Two-way ANOVA results for pollutant inhibition ratios.

Pollutant	Biochar Type (*p*-Value)	Biochar Dosage (*p*-Value)
TVOC	<0.0001	0.0190
H_2_S	<0.0001	0.0296
SO_2_	<0.0001	<0.0001
NO_X_	<0.0001	0.0220

**Table 8:** Number of GC–MS peaks retained after screening.

Item	AR	WB-AR-2	WSB-AR-2	CSB-AR-2
Raw TIC peaks	221	204	160	201
Valid peaks	170	163	145	167
Unique tentative assignments	141	131	113	132

## Data Availability

The original contributions presented in this study are included in the article. Further inquiries can be directed to the corresponding author.

## Supplementary Materials

The following supporting information can be downloaded at: https://www.mdpi.com/article/10.3390/polym18212689/s1, Figure S1: SEM micrographs of WB, WSB, and CSB at magnifications of 1000× and 5000×; Table S1: BET fitting parameters for the three biochars.
