# Supplementary material for: Systematic Investigation into Biochar-Mediated Suppression of Asphalt Rubber Fumes: Inhibition Mechanism, Pollutant Profiles, and Binder Performance
Source: Polymers (Basel). 2026 Oct 29;18(21):2689. doi: 10.3390/polym18212689 (PMC13766020; doi:10.3390/polym18212689)
Supplement: Supplementary file 1 [file polymers-18-02689-s001.zip › polymers-4562933-supplementary.pdf]

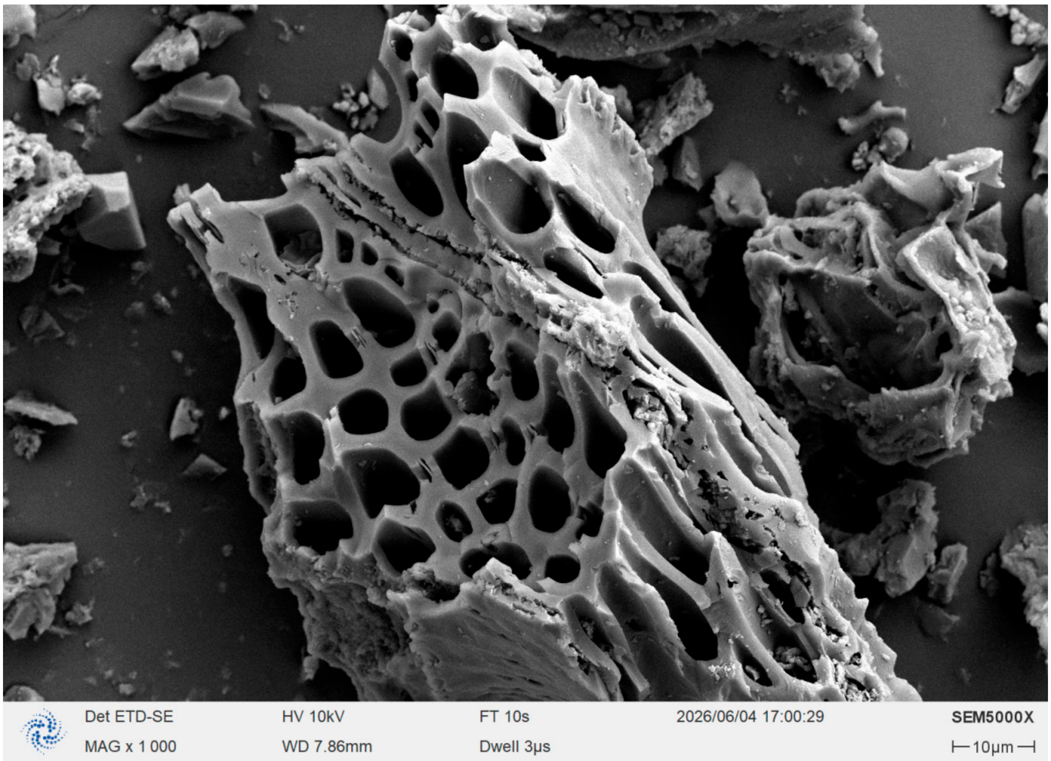
Det ETD-SE
MAG x 1 000
HV 10kV
WD 7.86mm
FT 10s
Dwell 3μs
2026/06/04 17:00:29
SEM5000X
10μm

WB

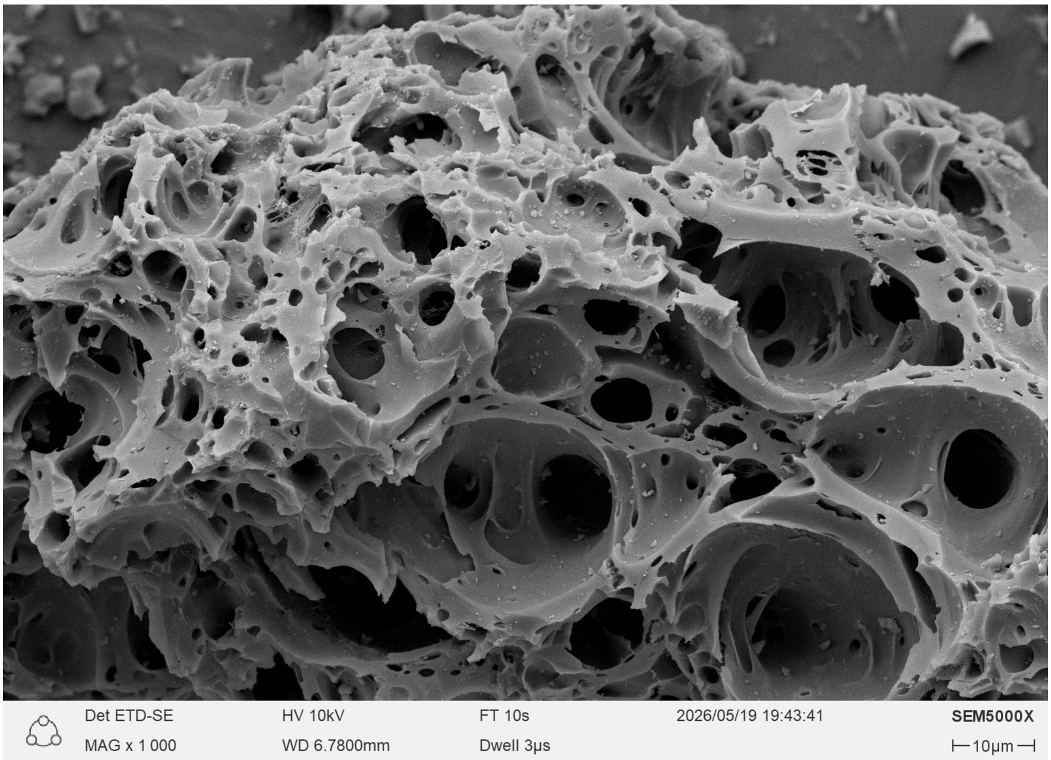
Det ETD-SE
MAG x 1 000
HV 10kV
WD 6.7800mm
FT 10s
Dwell 3μs
2026/05/19 19:43:41
SEM5000X
10μm

WSB

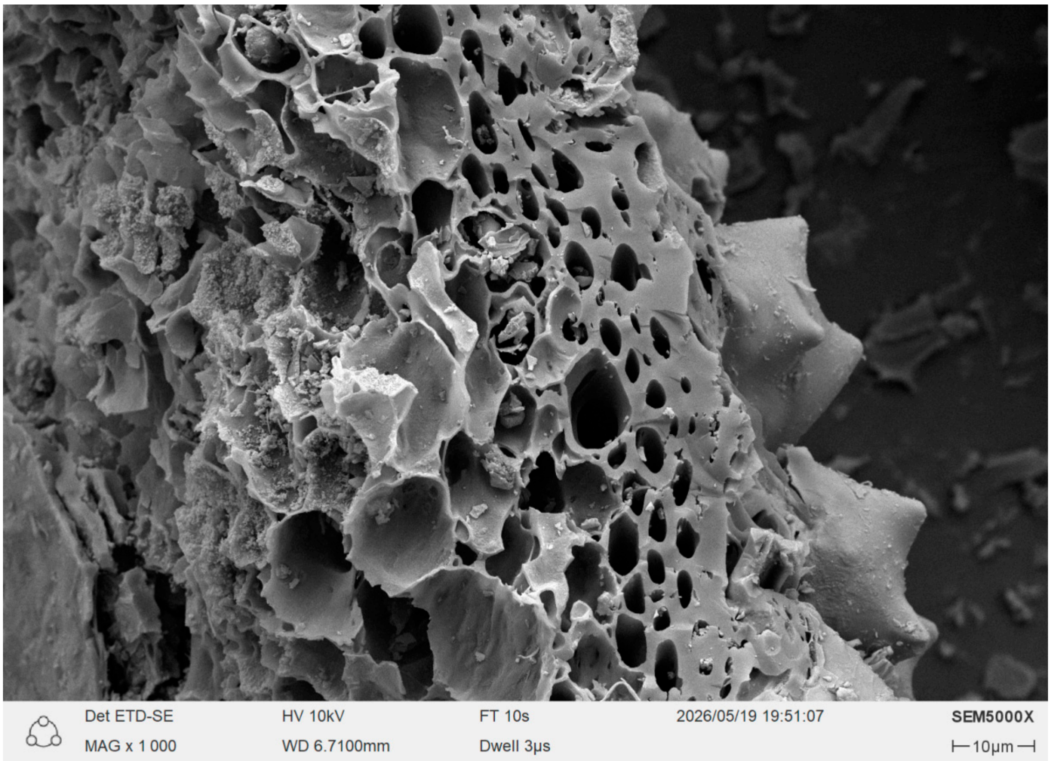
Det ETD-SE
MAG x 1 000
HV 10kV
WD 6.7100mm
FT 10s
Dwell 3μs
2026/05/19 19:51:07
SEM5000X
10μm

CSB

(a) ×1000

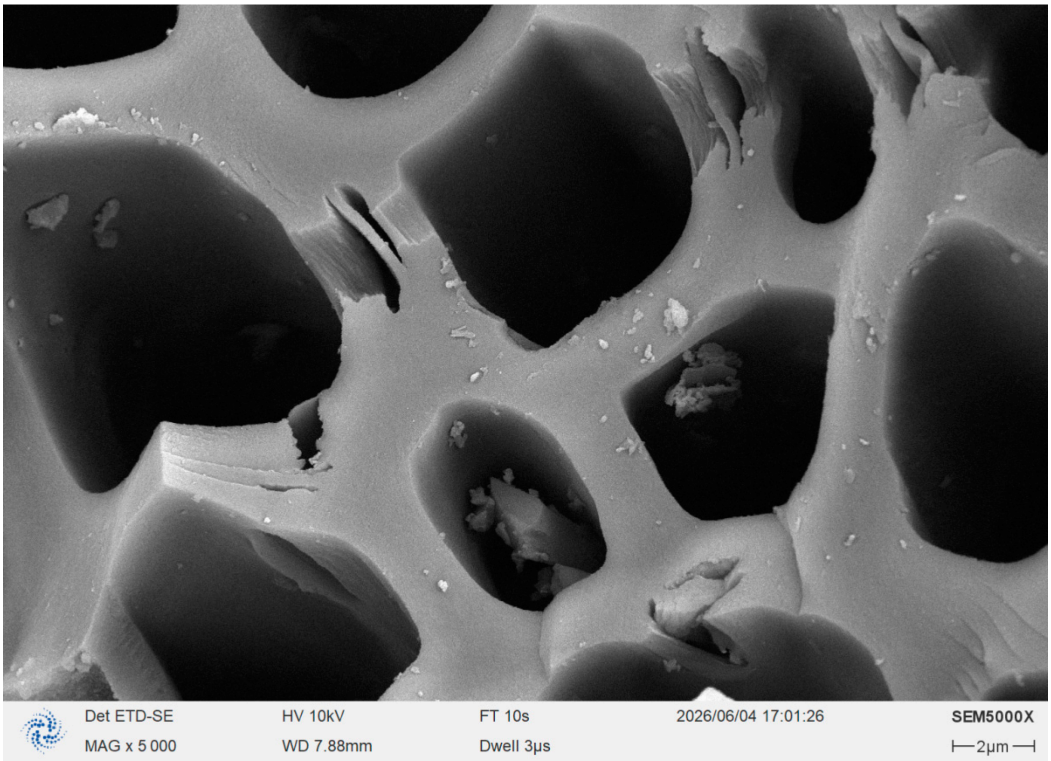
Det ETD-SE
MAG x 5 000
HV 10kV
WD 7.88mm
FT 10s
Dwell 3μs
2026/06/04 17:01:26
SEM5000X
2μm

WB

(b) ×5000

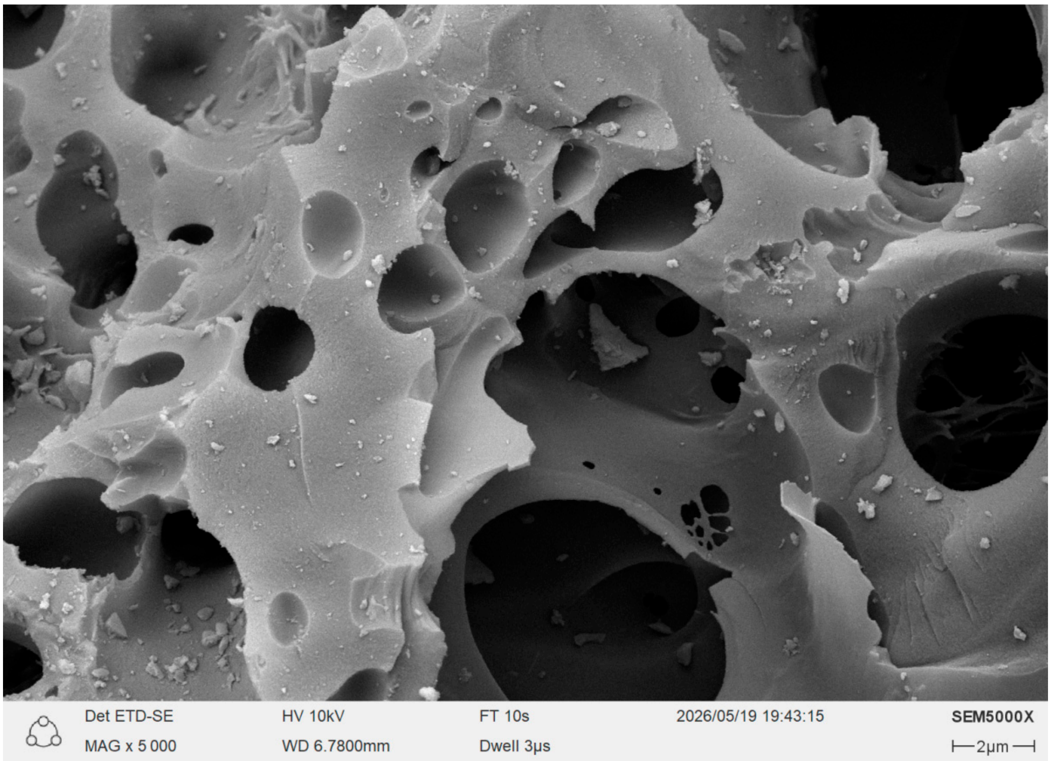

WSB

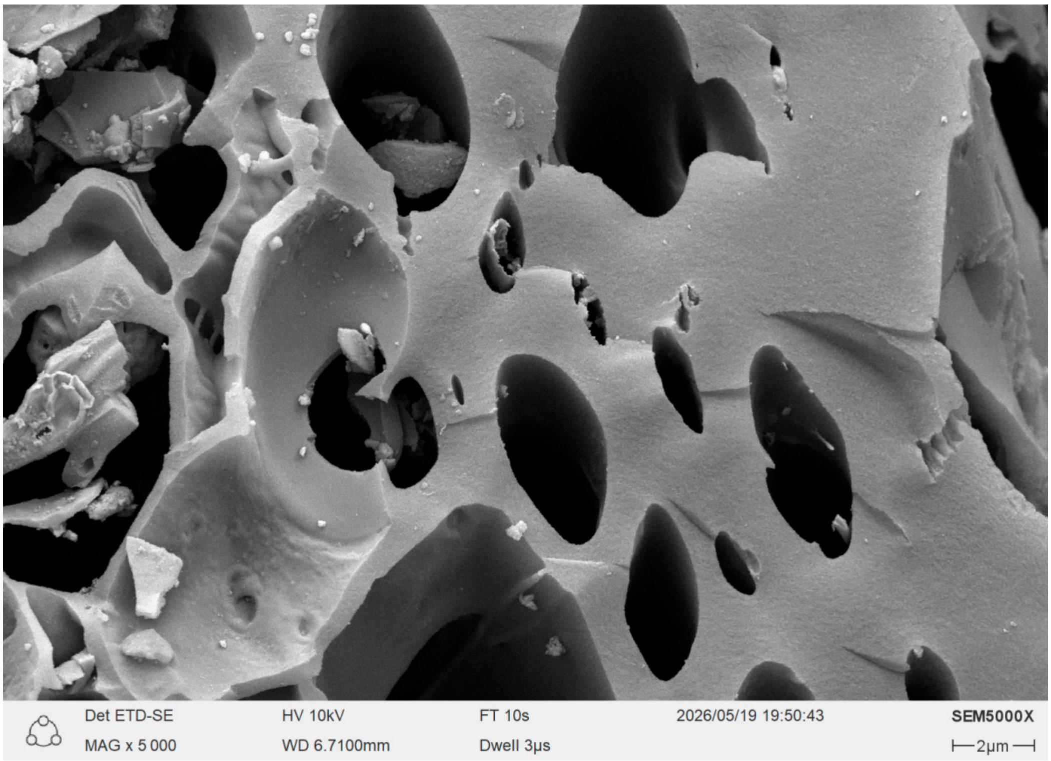

CSB

(b) ×5000

Figure S1. SEM micrographs of WB, WSB, and CSB at magnifications of 1000× and 5000×.

Table S1. BET fitting parameters for the three biochars.

Parameter	WB	WSB	CSB
BET fitting range (P/P_0)	0.0757–0.2276	0.0099–0.0997	0.1006–0.2523
R^2	0.99988	0.99668	0.99996
Positive C constant	Yes	Yes	Yes
Monotonically increasing n(1−P/P_0)	Yes	Yes	Yes
Monolayer pressure within fitting range	Yes	Yes	Yes
